# Computational approaches for isoform detection and estimation: good and bad news

**DOI:** 10.1186/1471-2105-15-135

**Published:** 2014-05-09

**Authors:** Claudia Angelini, Daniela De Canditiis, Italia De Feis

**Affiliations:** 1 Istituto per le Applicazioni del Calcolo, CNR, Naples, Italy; 2 Istituto per le Applicazioni del Calcolo, CNR, Rome, Italy

## Abstract

**Background:**

The main goal of the whole transcriptome analysis is to correctly identify all expressed transcripts within a specific cell/tissue - at a particular stage and condition - to determine their structures and to measure their abundances. RNA-seq data promise to allow identification and quantification of transcriptome at unprecedented level of resolution, accuracy and low cost. Several computational methods have been proposed to achieve such purposes. However, it is still not clear which promises are already met and which challenges are still open and require further methodological developments.

**Results:**

We carried out a simulation study to assess the performance of 5 widely used tools, such as: CEM, Cufflinks, iReckon, RSEM, and SLIDE. All of them have been used with default parameters. In particular, we considered the effect of the following three different scenarios: the availability of complete annotation, incomplete annotation, and no annotation at all. Moreover, comparisons were carried out using the methods in three different modes of action. In the first mode, the methods were forced to only deal with those isoforms that are present in the annotation; in the second mode, they were allowed to detect novel isoforms using the annotation as guide; in the third mode, they were operating in fully data driven way (although with the support of the alignment on the reference genome). In the latter modality, precision and recall are quite poor. On the contrary, results are better with the support of the annotation, even though it is not complete. Finally, abundance estimation error often shows a very skewed distribution. The performance strongly depends on the true real abundance of the isoforms. Lowly (and sometimes also moderately) expressed isoforms are poorly detected and estimated. In particular, lowly expressed isoforms are identified mainly if they are provided in the original annotation as potential isoforms.

**Conclusions:**

Both detection and quantification of all isoforms from RNA-seq data are still hard problems and they are affected by many factors. Overall, the performance significantly changes since it depends on the modes of action and on the type of available annotation. Results obtained using complete or partial annotation are able to detect most of the expressed isoforms, even though the number of false positives is often high. Fully data driven approaches require more attention, at least for complex eucaryotic genomes. Improvements are desirable especially for isoform quantification and for isoform detection with low abundance.

## Background

Gene transcription represents a key step in the biology of living organisms. Several recent studies, including [1,2], have shown that, at least in eukaryotes, a large fraction of the genome is transcribed and almost all the genes (more than 90% of human genes) undergo alternative splicing. The discovery of the pervasive nature of eukaryotic transcription, its unexpected level of complexity - particularly in humans - and its accurate quantification are helping to have a deep insight into biological pathways and molecular mechanisms that regulate disease predisposition and progression [3].

The main goal of the whole transcriptome analysis is to identify, measure, characterize and catalogue all expressed transcripts within a specific cell/tissue - at a particular stage and condition - in particular to determine the precise structure of genes and transcripts, the correct splicing patterns, their abundances, and to quantify the differential expressions in both physiological and pathological conditions.

Thanks to pioneer works of [4-6] that showed, among others, the potential of high-throughput mRNA sequencing (RNA-seq) and the development of efficient computational tools [7-9] to analyse such a data, RNA-seq has quickly become one of the preferred and most widely used approaches for discovering new genes and transcripts and for measuring transcript abundance from a single experiment (see [10,11] for reviews). To date, RNA-seq experiments have been successfully used in a wide spectrum of researches, offering tremendous benefits with respect to those previous approaches, such as microarrays, and also creating many challenges from both experimental and data analysis perspective [12].

In particular, to fully benefit of RNA-seq data, the following (strongly connected) computational challenges must be faced: **i)** Transcriptome reconstruction or isoform identification **ii)** Gene and Isoform detection (on/off) **iii)** Gene and Isoform quantification (expression level in terms of either FPKM or read-count) **iv)** Gene and Isoform differential expression

Points i)–iii) are aimed to provide a full characterization of the transcriptome of a given sample, with ii) and iii) often combined into a simultaneous step, where some parsimonious strategies are employed to deal with the high number of candidate isoforms. Point iv) is carried out to compare samples across different physiological and pathological conditions. To face these challenges, several computational methods have been proposed [13,14] and open-source software packages are available. However, despite the connection among the previous points, most of the available computational methods attempt to face each point independently. Therefore, sophisticated pipelines are built in order to provide a comprehensive answer (see the Tuxedo pipeline [15] as a remarkable example). Anyway, the choice of the best method to use for a specific dataset, the best parameter tuning and the expected performance are not clear to a beginner user. In particular, methods often require several additional parameters that are not easy to understand and choose. Assessing the best combination is very difficult and time consuming. In most cases the choice is done in a subjective way, partially driven by prior knowledge of the structure of the genome under analysis and by some heuristic considerations, rather than using an objective and general approach. Therefore, most users are often confused and tend to use default values.

Recently, few independent studies have been devoted to compare the performance of computational methods for detecting differential expression under a wide type of settings, see for example [16-18]. For what concerns points i)–iii) limited comparisons were carried out within the same paper that describes the proposed method [19-23]. However, to the best of our knowledge, no independent comparison was available until the recent study of [24], conducted almost simultaneously to our study.

Goals of the present paper are to illustrate the results of a detailed comparison of five widely used tools, namely *CEM*[23], *Cufflinks*[20], *iReckon*[22], *RSEM*[19] and *SLIDE*[21], to provide a discussion about expected results, and to assess which promises are already met and which challenges are still open and require further methodological developments. Even though, at least for data driven approaches, our conclusions are similar to those achieved by [24], the way to carry out our analysis and the way to compare the methods are different. Therefore, this study can be viewed as a complement to [24]. Specifically, we assessed the particular improvements that may be obtained by using annotation.

In the following section, we briefly review some of the most widely used tools for isoform reconstruction and quantification in organisms for which a reference genome is available. When the reference genome is not available (or the user does not want to use it) such methods cannot be used and more computational demanding assembly strategies have to be taken into account instead, see for example [25,26] or more in general [24]. Subsequently, in Methods section we describe the approach considered to build the comparisons and provide the rationale about the compared methods, their parameters and modes of usage. Comparisons are mainly carried out for simulated paired-end reads (PE) with different through-put and read-lengths, since they represent the state of the art of most current experiments. However, for completeness we also implemented a limited simulation study using single-end reads (SE) at the same depth. All methods were mostly used with their default parameters, without attempting any internal parameter optimization to improve their performances, mimicking the expected usage of a non expert scientist in the analysis of RNA-seq data.

Methods were compared under different experimental scenarios, assuming the availability of complete annotation, incomplete annotation and absence of annotation. Moreover, whenever possible, such scenarios were combined with three different modes of action that account for different strategies in the considered algorithms. In the first mode, the inference is limited only to those isoforms that are present in the annotation. In the second mode, the annotation is used as a guide to identify other possible transcripts. In the third mode, all inference is fully data driven. We observe that the case of complete annotation (combined with inference limited to handle only transcripts contained therein) represents an ideal case, that allows us to evaluate the performance of each method in detecting presence of isoforms and to quantify their expression when everything else in known. This situation is rarely met given our current knowledge of Biology. However, it can be considered as a limit case since, in the near future, it will be possible to work with almost complete annotations, thanks to the output of large international projects such as ENCODE (Encyclopedia of DNA Elements) [27], at least for widely studied organisms such as the Human one. The case of incomplete annotation (combined with an inference driven by the provided annotation) illustrates the realistic case in current studies, where previous projects have disclosed most information. However, data emerging from the literature show that such information represents only partial knowledge. Finally, the full data driven approach is necessary when studying novel sequenced organisms for which no previous information is available (or the user does not want to use the annotation explicitly). All comparisons in [24], except iReckon and SLIDE, were carried out fully data driven.

In Results and discussions section, we illustrate the results of our comparative study on two different experimental set-ups. The first models small genomes, while the second models large genomes. In particular, we compare recall and precision given a set of truly expressed isoforms and we evaluate the quality of the abundance estimates. Also with respect to this point, our method differs from [24]. In our case the truth is based on simulated data, about which we have a complete knowledge in terms of truly expressed isoforms and expression levels. Conversely, in [24] the comparison was carried out on a real dataset for which the same information was not available. In that case the aim was to benchmark data driven approaches in recovering the gene annotation, without taking into account whether the retrieved isoforms were present in the sample or not. Finally, Conclusions section summarizes all our evaluations.

### An overview on computational methods for isoform identification and quantification

The classical pipeline for isoform detection and estimation consists of the following three logical steps. First, the reads are aligned to the reference genome. Subsequently, candidate isoforms are either identified or are directly provided by the user through an annotation file. Finally, the presence and the abundance of each isoform are (either independently or simultaneously) estimated. We refer to [13,14] for detailed reviews of the existing algorithms and software. Alternatively, it is also possible to use methods, such as [26], that assemble reads in longer fragments that constitute the transcriptome, and then use methods for quantifying the abundance of inferred transcripts. Assembly methods are based on local alignment and graph theory and are similar in spirit to those methods used to assemble genomes. Such methods are potential very interesting for detecting de-novo isoforms. However, the comparison of such approaches with aligned based algorithms is out of the scope of the current work.

RNA-seq alignment can be performed by a series of devoted tools such a [28-32], that allow to map both reads to the reference genome without large gap (i.e., exon-body reads) and reads with large gap in terms of genomic coordinates that span exon-exon junctions (i.e., splice-junctions reads). Since the aim of this paper is to compare isoform estimation/detection procedures, we chose for the alignment step Tophat2 [29] (version 2.0.7) and we refer to [18,31,33,34] for comparisons on different algorithms. The choice of Tophat2 is motivated by the fact that the analysed tools suggest it, or its previous version [28], as aligner. Nevertheless, in general these methods only require the user to provide an alignment file. Therefore, any of the existing RNA-seq mappers can be used. The ability of an aligner to properly map the junction reads is important since false negative junctions may prevent the possibility of reconstructing some isoforms, while false positive junctions can lead to false isoform identification. We also note that some methods, for example [35], align reads to the transcriptome to better map the (known) splice junctions. Others, such as [29], implement hybrid approaches using both transcriptome and genome.

Once the read alignment has been performed, the inference can be carried out at different biological levels. Quantification of multiple isoforms is more complicated than the single event one (i.e., exons, junctions or genes), since different isoforms of the same gene (or that insist on the same genomic locus) share great part of the sequences from common exons and junctions. Moreover, identification and quantification problems are affected by both positional and sequence content biases present in RNA-seq data and by several other -still not fully understood- sources of experimental biases. The differences among the methods mostly depend on the way they model reads and the way they account for the different sources of biases.

In principle RNA-Seq data (i.e. observed coverage and splice-junction) can be modeled as a linear combination of isoforms. Therefore, the problem can be seen as a deconvolution problem [36,37] with expression levels as weights and isoforms as convolution kernels. Under such formalism, the isoform expression can be estimated either by using the “maximum likelihood principle” or by using similar statistical optimizations. Unfortunately, the design matrix that describes the isoform structures is unknown (or at least not completely known) and potentially very large. Therefore, the problem can be treated as a two steps procedure where, first, a set of candidate isoforms is identified, then the inference is made on such a set. The isoform identification step is crucial since the rest of inference is carried out on the basis of this result. On the other hand, it is also possible to perform the two steps simultaneously, see [38]. Moreover, because of the large number of candidate isoforms, the problem becomes ill-posed. Therefore, some penalties have to be used to encourage sparse solutions and avoid data over-fitting.

For instance, the very famous Lasso-type penalty was used in [21,39]. This penalty is sub-optimal since it does not take into account that all abundances are non negatives and their sum is constrained. The penalty in [22] is one attempt to explicitly use such constraints and reinforce the sparsity. In other cases, see for example [20] the sparseness is achieved using post-filtering steps to reduce the number of candidate isoforms.

It should be noted that several methods proposed for studying isoforms [36,40,41] do not perform the identification step explicitly. Indeed, they require the user to provide such a-priori knowledge. This is usually done either in terms of annotation file (in.GTF or.BED format) that can be downloaded from some database (e.g., [42]) or as a preliminary result of some tools for transcript reconstruction, such as in [43]. In this context, the inference is often limited to the easier problem of quantifying only those isoforms that are contained in the annotation rather than identifying novel isoform structures. Despite the availability of several methods that allow both isoform reconstruction and quantification, we consider useful to consider those approaches since, when the annotation will become complete, they can turn back in competition. Moreover, providing them a list of candidate isoforms obtained from some assembly procedures, such methods claim to return accurate quantification. An example of such idea is given by RSEM [19] that is now used as quantification step combined with Trinity [26].

More in general, for methods performing the identification step, isoform reconstruction can be carried out by using two other philosophical approaches. In the first one, the algorithm is driven by an annotation (that represents the available information at state of the art). In the second case, all isoforms are reconstructed ab initio (or fully data driven), mainly using graph theory. These models must be used in combination with some (heuristic) approaches in order to make the graph optimization feasible due to the large number of potential transcripts coming from a splicing graph. Moreover, it often occurs that a same method can use different rationales according to the way it is used, see [20,23,43].

## Methods

In order to evaluate and compare the performances of the proposed methodologies, we used simulated data for which the true isoform structures and abundances are known.

Few RNA-Seq simulators have been proposed in the last years (BEERS Simulator [31], RSEM Read Simulator [35], RNASeqReadSimulator [44]). In this work we used Flux Simulator [45] (available at http://sammeth.net/confluence/display/SIM/Home), which is a tool able to model most of the experimental steps. Indeed, it takes into account reverse transcription, fragmentation, adapter ligation, PCR amplification, gel segregation and sequencing.

We ran Flux Simulator with the default file of parameters suggested for H. Sapiens (see Section 5.2 of the user manual at http://sammeth.net/confluence/pages/viewpage.action?pageId=786691) where we only changed the number of molecules (*NB*_*MOLECULES*), the number of reads (*READ*_*NUMBER*) and the read length (*READ*_*LENGTH*) to achieve the desired sparsity. We used the error model for reads of length 76 bp provided in the software, as suggested in the user manual, because the simulator scales the error profile to the chosen read length not explicitly supported by the model.

As output Flux Simulator returns a.pro file containing for each transcript the number of simulated reads originating from it and its length in bp. Therefore, for each transcript the “true” abundance was evaluated in terms of FPKM (Fragments Per Kilobase of transcript per Million of mapped fragments). Transcripts not originating any simulated read were considered as not expressed.

### Simulation scheme

Human genome (Hg19, UCSC) was considered as reference organism and release 69 annotation file was downloaded from Ensembl database [46]. For simplicity, we took into account only transcripts coding for proteins (i.e., 142692 potential transcripts in total).

Two experimental set-ups were simulated using Flux Simulator: **Set-up 1**, in which all transcripts from chromosome 1 were considered as Complete Annotation (CA), i.e., CA contains 13123 transcripts; **Set-up 2**, in which we considered a subsample of 85615 transcripts uniformly sampled from the list of all protein-coding transcripts as CA (i.e., CA contains 85615 transcripts). These scenarios were used to investigate the capability of the compared methodologies to deal with “small” and “large” genomes.

For **Set-up 1** Flux Simulator generated a large dataset of (strand-specific) PE reads of 100 bp per side and a set of 3726 transcripts with positive FPKM; for **Set-up 2** it generated a dataset of (strand-specific) PE reads of 75 bp per side and a set of 17032 transcripts with positive FPKM. Fastq files of reads underwent to a filtering process to remove those pairs that had one of the two sides smaller than 100 bp in **Set-up 1**, and one of the two sides smaller than 75 bp in **Set-up 2**, leading to a number of 31177152 PE fragments for **Set-up 1** and to a number of 74365564 PE fragments for **Set-up 2**.

To investigate the depth effect, in **Set-up 1** the simulated fragments were sub-sampled to obtain six subsets of cardinality 20M, 10M, 5M, 1M, 0.5M and 0.25M, where M stands for 10^6^ reads. To study the read-length effect, for each of the six subsets the reads were trimmed to obtain analogous sets of PE fragments of length 75 bp and 50 bp per side. Finally, to account for the library type effect, for each of the six sets of PE of 100 bp per side, only the left-mate reads were retained to obtain analogous sets of SE reads of length 100 bp. Analogously, in **Set-up 2** the dataset of PE reads was sub-sampled to obtain a subset of cardinality 60M, from which a second set was generated by trimming the reads at 50 bp.

Summarizing, overall eighteen PE datasets and six SE datasets were obtained under **Set-up 1** and two datasets under **Set-up 2**. Set-up 2 was also analyzed for depths of 40M and 20M (both at 75 bp and 50 bp). However, such results are not showed here for the sake of brevity.

To investigate the ability of different methods for transcript identification at different abundance levels, isoforms were divided in high, medium and low expression classes, where (analogously to iReckon) the low class is given by the isoforms whose true expression belong to the lower 5% of the FPKM distribution, the high class by isoforms with expression larger than the 74% of the FPKM distribution, the remaining ones representing the medium class.

For the sake of completeness, we also generated an Incomplete Annotation (IA). IA was obained from the corresponding CA selecting 70% of the annotated transcripts (i.e. 9186 in **Set-up 1** and 59930 in **Set-up 2**). In **Set-up 1**, IA was aimed to mimic a normal condition where most of the not annotated isoforms are present at low abundance in the RNA sample. Therefore, IA contains about 70*%* of the non expressed transcripts and the remaining 30*%* of expressed transcripts. In particular, the highly expressed transcripts represent 93% of the true high class, the moderately expressed transcripts represent the 64% of the true medium class and the lowly expressed transcripts represent only the 30% of the true low class. On the contrary, in **Set-up 2**, IA was obtained by randomly sampling the 70% of isoforms from the corresponding CA, regardless of their expression, to mimic the situation where tissue specific conditions or pathologies can alter the normal expression profile and produce novel transcripts that are present at any expression level.

### Read alignment

PE reads of each dataset were aligned to the human reference genome by using TopHat2 [29] with option **–library-type** fr-secondstrand turned on to benefit of the strand information of the simulated reads.

In particular, TopHat2 can be used with option **-G** turned on (i.e., by adding -G annotation.gtf to the command line). In this case, first, TopHat2 extracts the transcript sequences and uses Bowtie2 to align reads to this virtual transcriptome. Then, only the reads that do not fully map to the transcriptome are mapped to the reference genome where potential novel splice sites are identified. The reads mapped on the transcriptome are converted to genomic mappings (spliced as needed) and merged with the novel mappings and junctions in the final TopHat2 output. By contrast, if the option is turned off (i.e., -G is not used), TopHat2 aligns the reads directly to the genome and it searches for junctions with a data dependent approach [28]. It is clear that providing the annotation file allows TopHat2 to better map the splice junctions and cover the entire exon body, and to reduce the number of FP junctions, see [29] for a more detailed discussion.

For the scope of our analysis, when the option -G was turned on, we ran TopHat2 alignment on each set of PE reads twice. The first time, we provided CA. The second time, we provided IA. We also ran TopHat2 with the option -G turned off. For each set of the SE reads in **Set-up 1**, we repeated the alignment with TopHat2 using the same scheme.

### Modes of action

In our study, the problem of isoform detection and quantification is analyzed under different modes of action: **Mode 1)** The method assumes that annotation is available and the algorithm is forced to quantify only those isoforms in the given annotation. Those isoforms that are not present in the annotation are set to zero. **Mode 2)** The method assumes that annotation is available, but it could be incomplete. Therefore, the algorithm uses the provided annotation as a guide in order to find potentially new isoforms. After that all potential isoforms are identified, their expressions are quantified. **Mode 3)** The method assumes that no information is available. Therefore, all potential isoforms are computed from the data. Then, their expressions are quantified.

It is important to note that the modes of action can be combined with the different type of available annotation. In particular, under Mode 1 and 2 we can further distinguish the case that the available annotation is CA or IA. The case of Mode 1 with CA represents an ideal situation, when everything else is known. Such scenario is rarely met given our current knowledge of Biology, but can be considered as limit case for studies in the near future. The case of Mode 2 with IA represents a realistic scenario in current studies. Indeed, it is true that previous projects have disclosed most information, but still data emerging from the literature shows that such information represents only partial knowledge. Therefore, IA in Mode 2 represents a more realistic situation. For the sake of completeness, we observe that the usage of the methods under Mode 1 with IA will not allow to recover the not-given transcripts. Analogously, all novel transcripts detected by any method in Mode 2 with CA will be false positives. In both cases we are aimed to evaluate how such drawbacks can affect the estimation of other isoforms. Finally, Mode 3 is considered to illustrate the expected results that one can obtain when studying novel sequenced organisms for which no previous information is available (or when the user does not want to use it) and all inference has to be carried out from the experimental data. Moreover, comparing our simulation scheme with the analyses carried out in [24], we observe that their results correspond to Mode 3 without annotation, except for iReckon and SLIDE that were used similar to our Mode 2.

**Figure 1:**
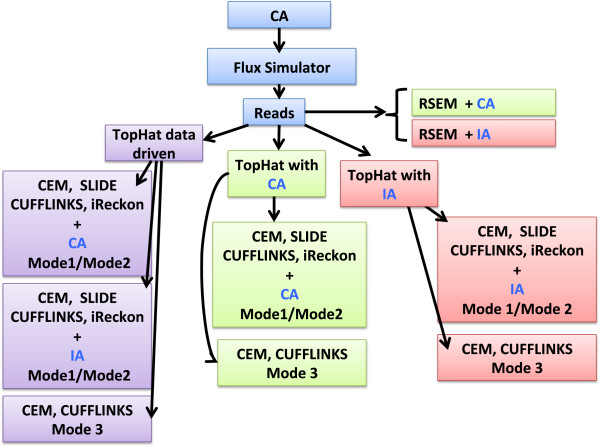
**Pipeline of the simulation.** Simulation workflow used both in Set-up 1 and Set-up 2. Complete annotation (CA) was given to Flux Simulator to generate strand specific PE reads (R). Reads were aligned on the reference genome using TopHat2. TopHat2 was independently used in three different ways (with CA, IA and without annotation). For each execution an alignment bam file was obtained. The alignment bam files were used as input for the compared methods. For each alignment file CEM and Cufflinks were used in Mode 1, 2 and 3; Slide was used in Mode 1 and 2, iReckon was used only in Mode 2. When providing annotation during the alignment, the same annotation was also used for Mode 1 and 2 (see green boxes for CA and pink boxes for IA). When the data driven alignment was carried out we further distinguished (in Mode 1 and 2) the cases with CA and IA, as annotation (see purple boxes). Since RSEM does not work with aligned reads, the output of Flux simulator was processed using CA or IA as annotation (depicted in a green and pink box, respectively).

Figure 1 illustrates the simulation pipeline built for the two experimental set-ups.

### Compared algorithms

In this paper, we assess the performance of five different methods: CEM, Cufflinks, iReckon, RSEM and SLIDE. All of them were used with mostly their default values and with modes of action illustrated in Table 1. We observe that Cufflinks and CEM can perform all modes of action, while the other methods only some of them. All methods where compared in **Set-up 1** for PE reads. All methods, except iReckon, were compared in **Set-up 1** for SE reads. All methods, except SLIDE, were compared in **Set-up 2**.

**Table 1:** Software used in the comparison

**Name**	**Version**	**Modes of action**	**Link**	**Publication**
TopHat2	2.07	CA/IA, no annotation	http://tophat.cbcb.umd.edu/	[28,29]
RSEM	1.2.3	1	http://deweylab.biostat.wisc.edu/rsem/	[19,35]
Cufflinks	2.0.2	1,2,3	http://cufflinks.cbcb.umd.edu/	[20]
SLIDE	May 7th, 2012	1,2	https://sites.google.com/site/jingyijli/	[21]
CEM	0.9.1	1,2,3	http://alumni.cs.ucr.edu/~liw/cem.html	[23]
iReckon	1.0.7	2	http://compbio.cs.toronto.edu/ireckon/	[22]

Table 1 shows detailed information for the software used during the study.

#### CEM

CEM [23] is a recent command line program written in C++ and Python developed by the authors of IsoLASSO [39] of which it constitutes a significant improvement. Its logic is very similar to the one of Cufflinks. Indeed the only required argument is the sam/bam alignment file. In this case, it executes Mode 3. The assembly problem is solved via a *connectivity graph*, which is more general than the *overlap graph* implemented in Cufflinks. By using optional parameter **-x**, the user can specify the annotation file (in BED format) and execute Mode 1 or Mode 2. If **–forceref** is turned on (i.e., -x annotation.bed – forceref), CEM will run in Mode 1. If the option **–forceref** is turned off (i.e., -x annotation.bed), the existing gene annotation will be incorporated into the estimation process as a guide from which CEM assembles new isoforms. Regardless of the action modes, the estimation of transcript abundance is carried out by minimizing a lasso penalized squared data-fit loss, where data-fit is given modeling the coverage in each segment as a Poisson distribution whose intensity is proportional to the mixture of abundances of the isoforms that insist on the same segment. With respect to this point, the main difference between CEM and its parent, IsoLASSO, consists in the algorithm used to perform minimization: CEM uses the Expectation-Maximization (EM) algorithm instead of quadratic programming. As a consequence CEM results by far more efficient than IsoLASSO, and overall one of the most efficient algorithm in terms of computational cost. No explicit parameter is available for strand specificity. CEM supports both SE and PE dataset.

#### Cufflinks

Cufflinks [20] is a popular software developed by the authors of TopHat and Bowtie and is part of the Tuxedo pipeline [15]. It is a command line tool, written in C++, where the only required argument is the sam/bam alignment file. In this case, it executes Mode 3. In particular, Cufflinks reduces the comparative assembly problem to a maximum matching problem in bipartite graphs and solves it by using the so call *overlap graph* approach. On the contrary, when using the optional parameters **-G** (i.e., -G annotation.gtf) or **-g** (i.e., -g annotation.gtf) the user can execute Mode 1 and Mode 2, respectively. Cufflinks was also used with the option **-u** turned on that allows an initial estimation procedure to assign more accurately those reads that mapped to multiple locations in the genome. Given the set of newly identified or annotated transcripts, for all modes of action, the transcript abundance is estimated via a Maximum Likelihood approach, where the probability of observing each fragment is modeled as a linear function of the transcript abundance that can originate the fragments. Because of linearity, the likelihood function has a unique maximum value that Cufflinks finds via a numerical optimization algorithm. Finally, we also observe that when the reads are aligned by TopHat by using strand-specific mode, Cufflinks will automatically treat data as strand-specific (otherwise library type has to be explicitly specified by the user). Cufflinks supports both SE and PE dataset.

#### iReckon

iReckon is a java software which implements the method presented in [22]. The input of iReckon are aligned reads, genome, annotation and reads themselves. Genome and annotation have to be provided in a house format obtained from Fasta and BED format after conversion in Savant [47]. The original reads and the genome files are necessary since after the construction of an enlarged transcriptome (i.e., all possible isoforms including pre-mRNA and isoforms with retained intron) from the mapped reads, the estimation is done by re-aligning the reads (using BWA 0.6.2, [48]) on the sequences of all possible isoforms. In principle iReckon could execute both in Mode 1 and Mode 2. Nevertheless, we used only Mode 2 (which is the iReckon’s default approach), due to a potential bug appearing when executing version 1.07 with the option **-novel 0** that was forcing the method in quantifying only transcripts in the annotation.

The main advantage of iReckon is that it directly models (and to date is the only one to have this feature) multiple biological and technical phenomena, including novel isoforms, intron retention, unspliced pre-mRNA, PCR amplification biases, and multi-mapped reads. iReckon utilizes the EM algorithm with a new non linear regularization penalty to accurately estimate the abundance of known and novel isoforms. The reason is that abundances are very similar to frequencies being non negative and summing to a normalization constant.

For large datasets and genomes, iReckon requires a large memory space and execution time. Most of the running time is due to the re-alignment step. No additional parameter is available for strand specificity. iReckon only supports PE dataset.

#### RSEM

RSEM [19] is a software package which implements the method originally presented in [35]. It is a command line program, written mainly in C++, with contributions in Perl and R. In contrast with previous methods, RSEM can only perform the estimation of isoform abundance given an annotation file, i.e. it can only work under Mode 1. As input data, RSEM requires either both the genome sequence and the annotation (in fasta and.GTF format) or the transcript sequences directly, and the read file. As first step, the reads are aligned directly to the transcript sequences using Bowtie (function **rsem-prepare-reference**); then abundances are estimated via an EM algorithm based on a generative statistical model that handle read mapping uncertainty (function **rsem-calculate-expression**). In particular, it uses an iterative process to fractionally assign reads to each transcript considering the probabilities of the reads being derived from each transcript and taking into account positional biases created by RNA-seq library-generating protocols. The interest in RSEM is that, although it can quantify only the known transcripts contained in the annotation file, if the annotation file includes potential novel transcripts or ab-initio estimates transcripts, the quantification can be performed as well. In this context it comes bundled with the Trinity software [26] where it is used to quantify the novel assembled transcripts. We used RSEM software with the **–strand-specific** option activated. RSEM supports both SE and PE dataset.

#### SLIDE

SLIDE (Sparse Linear modeling for Isoform Discovery and abundance Estimation) is a software mainly written in Python (with some R scripts) that implements the statistical method described in [21]. It can be used either in Mode 1 (**–mode estimation**) and in Mode 2 (**–mode discovery**). In particular, in Mode 2, SLIDE defines all the possible 2^*n*^−1 transcripts obtained by enumerating the *n* sub-exons of each gene.

In contrast to the other methods, SLIDE does not work on empirical read coverage, but it associates to each PE fragment four genomic locations corresponding to the starting and ending position of its 5’ and 3’ reads, respectively, and converts such positions into four sub-exon indexes. Then, it computes the fraction of pairs whose genomic locations span a given bin (i.e., a combination of four given sub-exons, for all sub-exon combination). Subsequently, it uses a linear model to approximate the observed bin proportions in terms of isoform proportions, which represent the parameters to be estimated. SLIDE estimates isoform abundance with a non negative least squares solution of a linear model. The design matrix models the conditional probability of sampling a PE read in a bin given that it comes from an isoform. A modified lasso type approach is used to limit the number of non null isoforms as well as to favor longer isoforms.

Finally, we observe that SLIDE has been mainly designed for relative small genomes. Therefore, the code has not been optimized. For this reason we compared its performance only on a small scale comparison (i.e., **Set-up 1**). In this case we considered both the higher confident output denoted, as “less”, and the larger one, denoted by “more”, similar to the original annotation. No explicit parameter is available for strand specificity. SLIDE supports both SE and PE dataset.

### Novel isoform matching

All methods used in Mode 1 (i.e. Cufflinks -G; RSEM; CEM - forceref and SLIDE - mode estimation) directly estimate FPKM of each isoform given in the annotation (CA or IA). Therefore, the association between the estimated value and the true value is straightforward. On the contrary, methods used in Mode 2 or Mode 3 allow to discover (new) isoforms. Therefore, their output need to be further processed in order to properly associate the inferred isoforms with the true ones. To this purpose, Cuffcompare v2.1.1 (which is part of the Cufflinks suite) was used to associate the output of any considered method (usually a gtf file) with the true CA. In particular, a ‘true’ isoform name was associated to an assembled isoform whenever a complete match of the intron chain was observed (i.e, Class Code ‘ =’ in <cuff_in>.tmap file, see user manual of [20] for details). Such level of transcript matching is quite stringent and could be relaxed, since in some cases we noticed that the match in Mode 2 and 3 was achieved at lower level of stringency (for example transcripts were contained in the true ones or other Class Codes were returned by Cuffcompare). However, the choice of using stringent match does not change the conclusions, but only the actual values of the performance indexes. The assembled transcripts which did not match an annotated one were classified as novel. In few cases, more assembled isoforms were associated to the same annotated isoform. Then, the estimated FPKM of the isoform was evaluated as the sum of all estimated FPKMs. Similarly to [24], for iReckon, transcripts with intron retention or unspliced events were not considered.

### Measures of performance

In order to measure the performance of the considered methods, we first evaluated their capability in isoform detection in terms of true positives (TP), false positives (FP) and false negatives (FN), then their accuracy in isoform estimation in terms of estimation error.

For isoform detection, the following indicators were computed: 

● **Recall** (aimed at measuring the fraction of truly expressed isoforms that is retrieved) defined as

(1) $$\text{recall}=\frac{\text{TP}}{\text{TP}+\text{FN}}=\frac{\left|\left\{\hat{\text{FPKM}}>0\right\}\cap \{\text{FPKM}>0\}\right|}{\left|\{\text{FPKM}>0\}\right|}.$$

● **Precision** (aimed at measuring the fraction of predicted expressed isoforms that are truly expressed) defined as

(2) $$\;\text{precision}=\frac{\text{TP}}{\text{TP}+\text{FP}}=\frac{\left|\left\{\hat{\text{FPKM}}>0\right\}\cap \{\text{FPKM}>0\}\right|}{\left|\left\{\hat{\text{FPKM}}>0\right\}\right|}.$$

where $\hat{\text{FPKM}}$ represents the estimated abundance of the isoform, *FPKM* is the true abundance and |*S*| stands for cardinality of set S. If *FPKM*>0 the isoform is truly expressed, while if *FPKM*=0 the isoform is not expressed, analogously for the estimated values. Obviously, recall is a measure of completeness and precision is a measure of accuracy. Recall was also evaluated on abundance classes (low, medium and high) defined as described in Section Simulation scheme.

As a global measure of performance we also considered the following 

● **F-Measure** defined as

(3) $$F=\frac{2*\text{precision}*\text{recall}}{\text{precision}+\text{recall}}.$$

For evaluating the accuracy in abundance estimation, we distinguished three cases and considered 

● **Estimation Error** (aimed at quantifying the FPKM retrieval accuracy) defined as

(4) $$\;\text{error}\;=\;\left\{\;\;\begin{array}{lll} E_{1}=\frac{\hat{\text{FPKM}}-\text{FPKM}}{\text{FPKM}} & \text{if} & \text{FPKM}>0\;\;\text{and}\;\;\hat{\text{FPKM}}>0\\ E_{2}=\hat{\text{FPKM}} & \text{if} & \text{FPKM}=0\;\;\text{and}\;\;\hat{\text{FPKM}}>0\\ E_{3}=\text{FPKM} & \text{if} & \text{FPKM}>0\;\;\text{and}\;\;\hat{\text{FPKM}}=0 \end{array}\right)\;\;,$$

where *E*_1_ quantifies the (relative) accuracy in estimating the expressed isoforms that the method is able to identify, *E*_2_ quantifies the abundances assigned to FP isoforms and *E*_3_ quantifies loss of expression for FN isoforms.

The last, but not the least, important thing to be considered is the computational cost. Since algorithms are implemented in different languages and can be used on different computational architectures that can benefit or not of parallelism, we believe that any precise quantification of computational cost would be not fair. Therefore, this point will be only discussed from qualitative point of view in Section Results and discussions.

## Results and discussions

In the following, we first compare the methods in terms of their capability in isoform detection, then in terms of their accuracy in isoform estimation. We stress that the goal of the comparison is not to make a rank list of the considered methods, but to underline global positive aspects, common weaknesses and open problems that might lead to over-optimistic conclusions about the performance of current methodology.

### Isoform detection

Here, we illustrate the results in terms of recall, precision and F-measure considering the following effects: type of alignment, modes of action, type of annotation, type of library, abundance level, read length and sequencing depth. In order to investigate such effects, the same figures have to be inspected several times evaluating different aspects each time. To facilitate such comparison, we first describe the general structure of the figures, then we focus the attention on some specific comparison.

**Figure 2:**
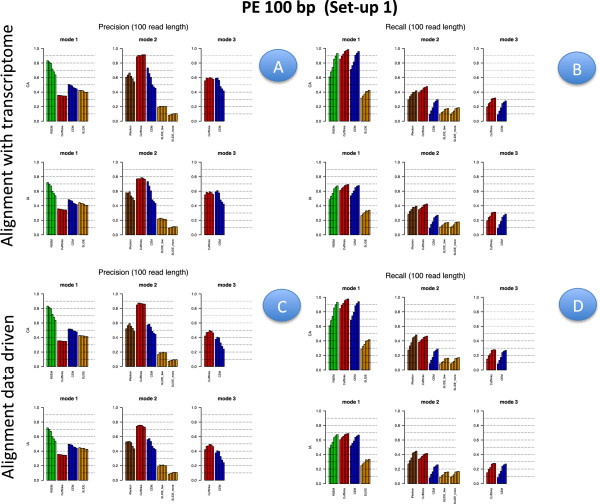
**Precision and Recall bar-plot in Set-up 1 for 100 bp-PE.** Panels **A** (upper left) and **B** (upper right) depict precision and recall bar-plots for the compared methods when the alignment is annotation driven. Panels **C** (bottom left) and **D** (bottom right) are analogous to Panels **A** and **B**, when the alignment is data driven. The figure refers to Set-up 1 and 100 bp-PE. Within each panel, the left column refers to methods used in Mode 1, middle column to methods used in Mode 2, right column to methods used in Mode 3; upper row represents results when the annotation is CA, bottom row is the analogous case when the annotation is IA. Different bars of the same colour for the same method and mode of action correspond to the different depth (i.e., from left to right 0.25M, 0.5M, 1M, 5M, 10M and 20M). When the alignment is annotation driven, the same annotation provided during the alignment was used for Mode 1 and 2.

**Figure 3:**
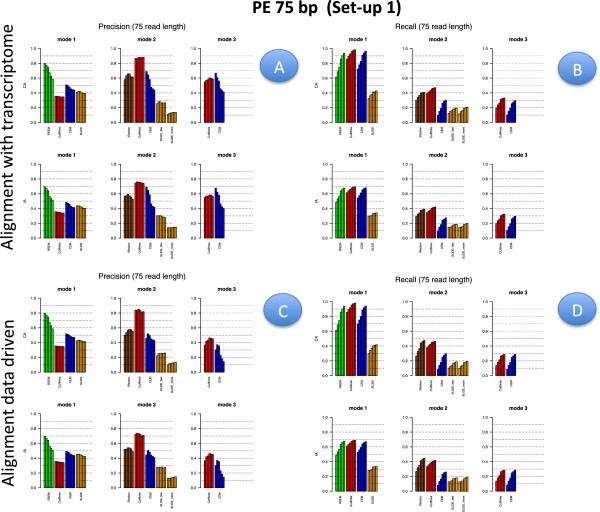
**Precision and Recall bar-plot in Set-up 1 for 75 bp-PE.** Analogous to Figure 2 but for Set-up 1 and 75 bp-PE.

**Figure 4:**
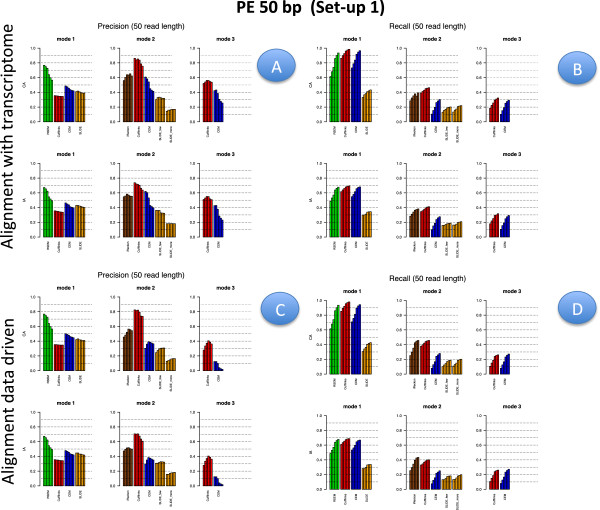
**Precision and Recall bar-plot in Set-up 1 for 50 bp-PE.** Analogous to Figure 2 but for Set-up 1 and 50 bp-PE.

**Figure 5:**
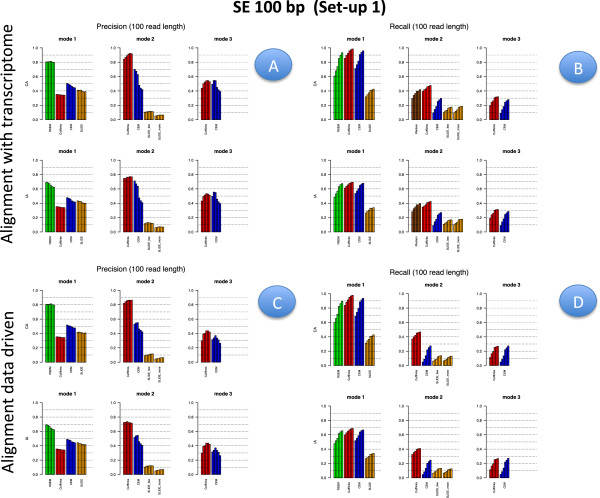
**Precision and Recall bar-plot in Set-up 1 for 100 bp-SE.** Analogous to Figure 2 but for Set-up 1 and 100 bp-SE.

**Figure 6:**
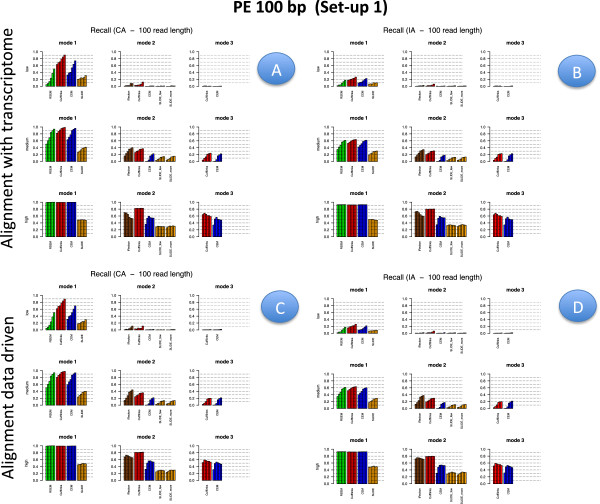
**Recall bar-plot versus isoform abundance in Set-up 1 for 100 bp-PE.** Panels **A** (upper left) and **B** (upper right) depict recall bar-plots versus isoform abundance for the compared methods when the alignment is annotation driven, CA and IA case respectively. Panels **C** (bottom left) and **D** (bottom right) are analogous to Panels **A** and **B**, when the alignment is data driven. The figure refers to Set-up 1 and 100 bp-PE. Within each panel, the upper row represents the recall observed for lowly expressed isoforms, middle row for moderately expressed isoforms and bottom row for highly expressed isoforms; left column refers to methods used in Mode 1, middle column to methods used in Mode 2, right column to methods used in Mode 3. Different bars of the same colour for each method and mode of action correspond to the different depth (i.e., from left to right 0.25M, 0.5M, 1M, 5M, 10M and 20M). When the alignment is annotation driven, the same annotation provided during the alignment was used for Mode 1 and 2.

**Figure 7:**
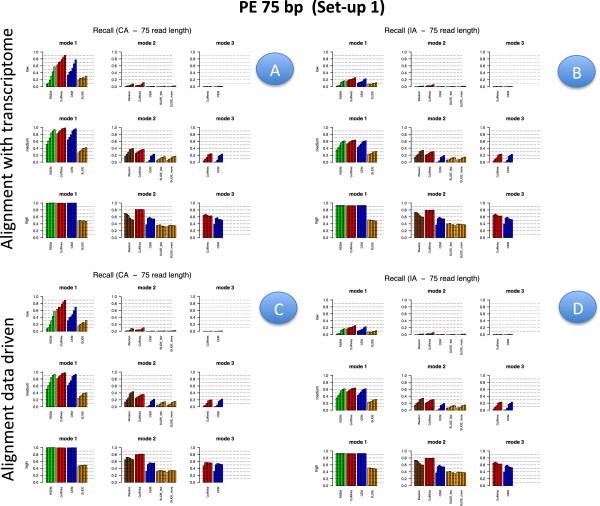
**Recall bar-plot versus isoform abundance in Set-up 1 for 75 bp-PE.** Analogous to Figure 6 but for Set-up 1 and 75 bp-PE.

**Figure 8:**
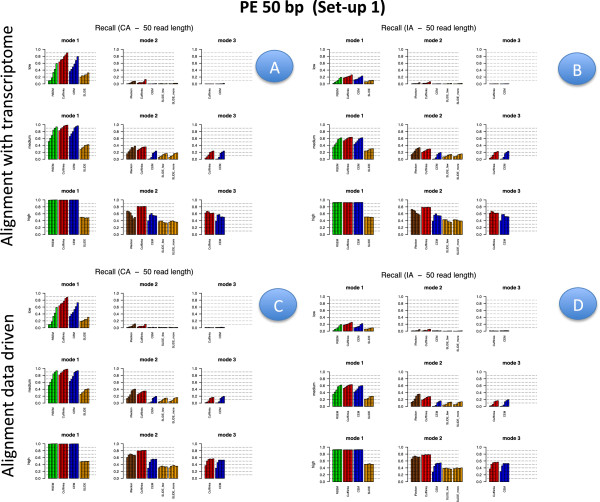
**Recall bar-plot versus isoform abundance in Set-up 1 for 50 bp-PE.** Analogous to Figure 6 but for Set-up 1 and 50 bp-PE.

**Figure 9:**
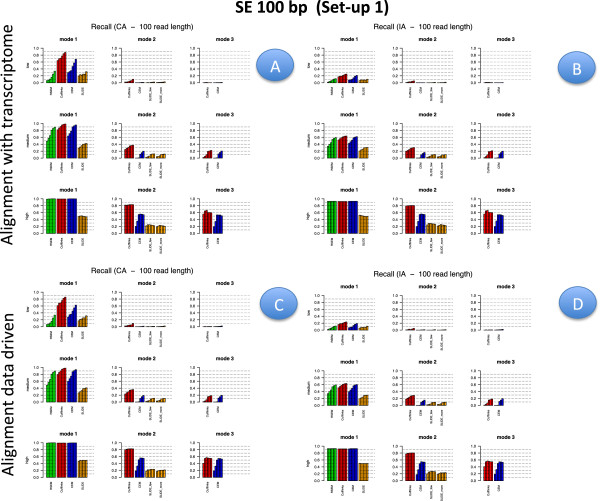
**Recall bar-plot versus isoform abundance in Set-up 1 for 100 bp-SE.** Analogous to Figure 6 but for Set-up 1 and 100 bp-SE.

Figures 2, 3, 4 and 5 illustrate results for precision and recall obtained in **Set-up 1** for libraries 100 bp-PE, 75 bp-PE, 50 bp-PE and 100 bp-SE, respectively. For each of these cases, recall is further expanded with respect to the level of abundance of the true isoforms and results are reported in Figures 6, 7, 8 and 9 in the same order. Finally, F-measure is illustrated in Additional file 1: Figure S1, Additional file 2: Figure S2, Additional file 3: Figure S3 and Additional file 4: Figure S4 in the same order for each of the four cases.

In Figures 2, 3, 4 and 5 results are visually depicted into four panels, A (upper left), B (upper right), C (bottom left) and D (bottom right). Panels A and B refer to the annotation driven alignment; panels C and D to the data driven alignment. Precision is depicted in panels A and C, while recall in panels B and D. Within each panel, the plot is divided in six sub-blocks according to the modes of action and the type of annotation. In particular, on the left blocks (Mode 1) there are RSEM, Cufflinks with the -G option turned on, CEM with the -forceref option turned on, SLIDE with -mode estimation. In the central blocks (Mode 2) there are iReckon, Cufflinks with the option -g turned on, CEM with the option -forceref turned off, SLIDE with -mode discovery. Finally, in the right blocks (Mode 3) there are only Cufflinks and CEM with all default options. Results obtained using CA/IA are depicted in the first/second row of each panel, respectively. In **Set-up 1** different bars for the same method and mode of action correspond to the different sequencing depth (0.25M, 0.5M, 1M, 5M, 10M and 20M, respectively). Dashed horizontal lines are added to facilitate comparisons among different cases. Since RSEM does not depend on the alignment strategy, for comparative purposes, panels A and C report the same precision and panels B and D the same recall, in correspondence of the same type of annotation for RSEM. Analogously, within each panel, methods in Mode 3 show the same precision and recall (within the upper and bottom rows of each panel), not depending on the provided annotation when the alignment is data driven.

Figures 6, 7, 8 and 9 have a similar organisation. In particular, CA is used in panels A and C, IA in panels B and D. Within each panel, the plot is divided in nine sub-blocks. Along the horizontal direction the organization is analogous to that of Figures 2, 3, 4 and 5, in the vertical direction recall is reported for the low, medium and high abundance classes, respectively.

Additional file 1: Figure S1, Additional file 2: Figure S2, Additional file 3: Figure S3 and Additional file 4: Figure S4 are also organized in four panels and illustrate the F-measure with respect the sequencing depth. Panels A and B refer to the annotation driven alignment; panels C and D to the data driven alignment. CA is used in panels A and C, IA in panels B and D. To better distinguish results among different modes of action, results in Mode 1 are shown with continuous lines, those in Mode 2 are reported in dashed lines, those in Mode 3 are reported in dotted lines.

For the sake of completeness, we also provide Additional file 5: Figure S5, Additional file 6: Figure S6, Additional file 7: Figure S7 and Additional file 8: Figure S8 showing the number of TP and FP obtained under **Set-up 1**, for the most extreme conditions PE vs SE, 20M vs 0.25M. The figures are again organized in four panels where panels A and B refer to the annotation driven alignment; panels C and D to the data driven alignment. CA is used in panels A and C, IA in panels B and D. Within each panel, the right bars (i.e., the one depicted in coral) show the number of FP and the left ones (depicted in aquamarine) the number of FN. An horizontal dashed line is added representing the number of truly expressed isoforms. Therefore, the difference between the number of FP and this line represents the number of FN.

**Figure 10:**
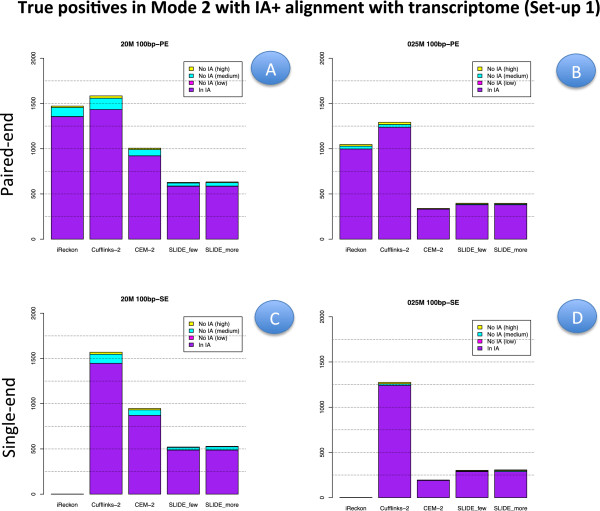
**True positive isoforms in Set-up 1, annotation driven alignment and Mode 2 with IA.** The panels depict the number of TP isoforms detected by methods in Mode 2 when IA is provided. They are divided in those that were already present in IA (bar in purple), and those not present in IA but retrieved by the methods. The latter are further divided in low, medium and high expression classes according to their true expression level. Panels **A** (upper left) refers to the case of 20M 100 bp-PE, Panels **B** (upper right) to 0.25M 100 bp-PE. Panels **C** (bottom left) and **D** (bottom right) are analogous to Panels A and B but for SE reads. All results are obtained with annotation driven alignment.

**Figure 11:**
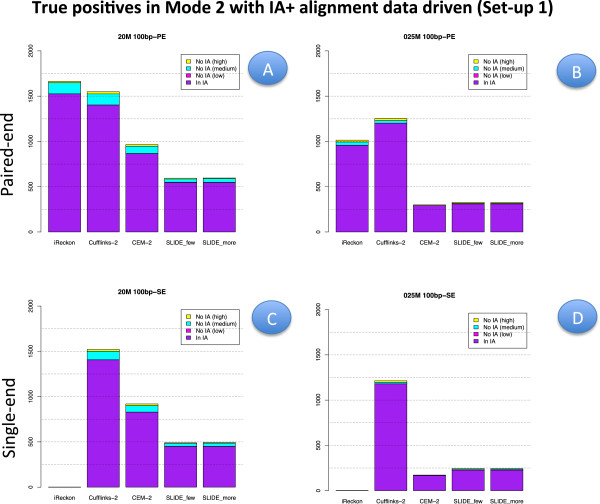
**True positive isoforms in Set-up 1, data driven alignment and Mode 2 with IA.** Analogous to Figure 10 but for Set-up 1 and data driven alignment.

**Figure 12:**
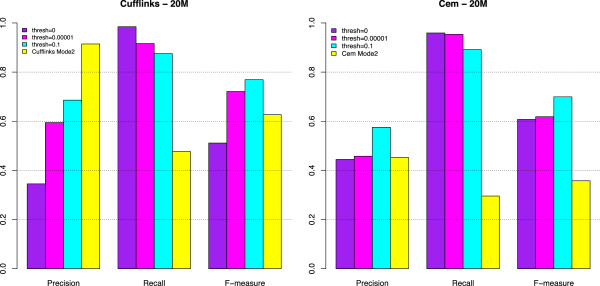
**Precision, Recall and F-measure with thresholds (Set-up 1).** Precision, Recall and F-measure for Cufflinks (left panel) and CEM (right panel). Within each set of bars, the first one (depicted in purple) reports the result for the corresponding method in Mode 1 (without any threshold), as depicted in Figure 2. The last one (depicted in yellow) refers to the same method in Mode 2, as depicted in Figure 2. The two central bars (depicted in magenta and cyan, respectively) refer to the method in Mode 1 when estimated isoforms with expression levels below 10^−5^ and 10^−1^, respectively, are set to zero. The figure refers to Set-up 1, to the case of 20M 100 bp-PE and annotation driven alignment with CA.

To provide a better insight into the capability of methods in Mode 2 (with IA) to identify those isoforms that are not provided in the annotation, Figures 10 and 11 show the number of TP (with the annotation driven alignment and with data driven alignment, respectively). In these figures, the number of TP is divided in those already present in IA (denoted IA) and those not contained in IA (denoted No IA). The latter are further divided according to the true expression level. Finally, Figure 12 illustrates the performance of Cufflinks and CEM (Mode 1) when a suitable threshold is applied to set to zero isoforms with very low estimated FPKM. The figure compares precision, recall and F-measure with the corresponding indexes observed for the same methods in Mode 2.

Additional file 9: Figure S9, Additional file 10: Figure S10, Additional file 11: Figure S11, Additional file 12: Figure S12, Additional file 13: Figure S13 and Additional file 14: Figure S14 show results for **Set-up 2**. In particular, Additional file 9: Figure S9, Additional file 10: Figure S10, Additional file 11: Figure S11, Additional file 12: Figure S12 are devoted to precision and recall. Additional file 13: Figure S13 is the analogous of Figure 12 and Additional file 14: Figure S14 is the analogous of Additional file 5: Figure S5, Additional file 6: Figure S6, Additional file 7: Figure S7 and Additional file 8: Figure S8.

All methods have been evaluated under the two set-ups, except SLIDE, that for the high computational cost has been evaluated only under **Set-up 1**, and iReckon, that was evaluated only for PE reads, since it does not support SE reads. In Figures 1, 2, 3, 4, 5, 6, 7, 8 and 9, Additional file 1: Figure S1, Additional file 2: Figure S2, Additional file 3: Figure S3 and Additional file 4: Figure S4 and Additional file 9: Figure S9, Additional file 10: Figure S10, Additional file 11: Figure S11 and Additional file 12: Figure S12, Cufflinks is coloured in red, CEM is coloured in blue, SLIDE is coloured in orange (when present), RSEM is coloured in green and iReckon is coloured in brown (when present).

RSEM shows relatively good performance when CA is provided, but its inference is limited to annotated transcripts (or to a list of potential transcripts that the user has to provide). On the other side, SLIDE shows worse performance than the others (in particular, in Mode 2) suggesting that it could be better suited for genome with lower complexity in terms of isoform structures.

Overall Set-ups 1 and 2 show similar qualitative results. The best F-measure in **Set-up 1** was about 0.78, in **Set-up 2** was 0.75. In both cases, the best performance was achieved by RSEM in the CA case.

Looking at Figures 2, 3, 4 and 5, precision rarely exceeds 0.6–0.7 and it is often below such values, meaning that all methods produce a quite large number of FP isoforms (remarkably, even when the true CA is provided and the methods are forced to work in Mode 1). Such drawback is also confirmed by the inspection of Additional file 5: Figure S5, Additional file 6: Figure S6, Additional file 7: Figure S7 and Additional file 8: Figure S8, where we often observe a high number of FP (compared to TP) for the same set-up. These poor results can be due to a non sufficiently strong penalty term (or to a not sufficient strong post-filtering step) that should keep to zero not significant isoforms.

The best precision is achieved by Cufflinks in Mode 2 with CA (at a price of lower power). Surprisingly, Cufflinks (Mode 1) shows a worse precision. However, we noticed that most of the isoforms detected as presents by Cufflinks (Mode 1) were estimated with extremely low expression. Therefore, if we filter out these isoforms, precision in Mode 1 and Mode 2 becomes comparable (see Figure 12, left panel), as expected. For example, by setting to zero all isoforms with estimated $\hat{\text{FPKM}}<10^{-5}$ the Cufflinks (Mode 1) precision increases to 0.59, without significantly reducing the recall (analogous behaviour with 10^−1^as threshold). Indeed, the F-measure of Cufflinks (Mode 1) with a threshold on the expressed isoforms becomes better than the Cufflinks (Mode 2) one, as expected. The tendency to produce a high number of FP with very low expression level was observed also for CEM (Mode 1), but with a much less pronounced effect (see Figure 12, right panel). The same effect occurs for the other conditions of depth, read length and library type (data not showed).

Overall recall is above 0.80 when methods are used in Mode 1 with CA (and above 0.6 with IA), while recall does not exceed 0.3 in Mode 3. Recalls in Mode 1 are mostly satisfactory (except for SLIDE). The actual observed recall values for Mode 2 and Mode 3 depend on the stringency of the match between newly identified isoforms and existing ones. However, the low recall values in Mode 3 show that the performance of methods in recovering the precise isoform structure is still not satisfactory.

Figures 6, 7 and 8 for PE and Figure 9 for SE datasets illustrate how well methods are able to detect highly expressed isoforms (remarkably, recall is mostly higher than 0.50 also for methods in Mode 3). At the same time they underline that the major problem arises in identifying lowly expressed isoforms (recall rarely exceeds 0.2 either in Mode 2 and Mode 3). In particular, we observe that lowly expressed isoforms are identified mainly in Mode 1 with CA. Methods in Mode 2 with IA are able to identify lowly expressed isoforms mainly if they were already present in IA. On the contrary, for moderately and highly expressed isoforms they are capable of detecting isoforms not provided in IA, see Figures 10 and 11. The figures show good results for Cufflinks and iReckon for medium and high expression classes, at high depth and regardless the alignment type.

The performance clearly drops down when decreasing the depth. In that case, it becomes almost impossible to recover isoforms that have not been provided in the annotation at the beginnig.

As mentioned above, **Set-up 2** bring us to analogous overall considerations, see Additional file 9: Figure S9, Additional file 10: Figure S10, Additional file 11: Figure S11, Additional file 12: Figure S12, Additional file 13: Figure S13 and Additional file 14: Figure S14.

#### Effect of alignment

In order to evaluate the effect of the alignment, we compared results obtained by the same method and mode of action across different alignment strategies, i.e., we investigate its effect on precision by comparing (in Figures 2, 3, 4 and 5) panel A versus panel C, and on recall by comparing panel B versus panel D, for **Set-up 1**. To evaluate the global effect on the F-measure, it is sufficient to carry out the analogous comparison for Additional file 1: Figure S1, Additional file 2: Figure S2, Additional file 3: Figure S3, and Additional file 4: Figure S4. Clearly, RSEM is not affected by the alignment, since it maps the reads directly to the transcriptome. Therefore, its results differ only with respect to CA and IA within panels A and B.

Overall comparisons show no appreciable difference in precision for Mode 1, a slight difference in Mode 2, where the precision increases along with the quality of mapping, and more remarkable improvements in Mode 3. Analogous results can be observed for recall. Moreover, both differences in precision and recall decrease when the sequencing depth increases. As global measure, we observe that F-measure for 20M 100 bp-PE is about 0.35 and 0.27, respectively, for Cuffinks and CEM in Mode 3 with reads aligned without any annotation; it becomes about 0.40 and 0.34, respectively, when the reads are aligned providing either IA or CA, see Additional file 1: Figure S1. To comment this effect, we inspected the alignment files and we observed that the main differences are in the number of mapped junctions. As an example, we observed that the dataset 20M 100 bp-PE identified 14500 junctions when CA was provided and 14479 junctions without annotation, with a very negligible loss due to the alignment. But, with 0.25M 100 bp-PE the number of mapped junctions was 9173 with CA and dropped down to 8047 without annotation. Analogously, for 20M 50 bp-PE, 14290 junctions were detected when CA was provided and only 12643 junctions without annotation; with 0.25M 50 bp-PE, the number of mapped junctions was 8241 with CA and dropped down to 5990 without annotation. The improved performance in Mode 3 can be explained by the fact that data driven methods can deeply benefit from the presence of informative junctions, even though current performances cannot be considered overall satisfactory.

The same conclusions apply for **Set-up 2**, comparing Additional file 9: Figure S9 and Additional file 10: Figure S10. In particular, in Mode 3, the F-measure increases from 0.18 of both Cufflinks and CEM in the case of data driven alignment up to 0.24 and 0.31 respectively in the case of CA based alignment (data not showed).

#### Effect of modes of action

In order to evaluate the effect of the modes of action, results have to be compared across the horizontal blocks of each panel of each figure, see Figures 2, 3, 4, 5, 6, 7, 8 and 9 for **Set-up 1** and Additional file 9: Figure S9, Additional file 10: Figure S10, Additional file 11: Figure S11 and Additional file 12: Figure S12 for **Set-up 2**. Moreover, we have to compare the continuous lines with respect to the dashed lines and the dotted lines in Additional file 1: Figure S1, Additional file 2: Figure S2, Additional file 3: Figure S3 and Additional file 4: Figure S4.

As expected, all methods in Mode 1 have better global performance than those in Mode 2 and perform significantly better than the methods in Mode 3. The latter ones are still a big challenge. Moreover, performance of methods in Mode 1 are much less affected by the depth and the read length, while still benefit of PE reads, if the coverage is not sufficiently high.

As previously mentioned, Figures 2, 3, 4 and 5 show that the precision cannot be considered fully satisfactory and the number of FP is high across all modes of action. In particular, Cufflinks (Mode 1) shows still poor precision. However, in this case, we already noted that most of the FP isoforms were estimated with a very low expression value and filtering out the low $\hat{\text{FPKM}}$s restores the performance, see Figure 12. From Figures 2, 3, 4 and 5, we observe that all methods in Mode 1 using CA have a very good recall, independently from alignment option. Methods in Mode 2 reach good/sufficient recall, except for SLIDE that shows the worst behaviour. In particular, it seems that SLIDE (Mode 2) does not take advantage of the annotation and behaves similarly to methods in Mode 3.

To fully understand the action mode effect, it is important to inspect Figures 6, 7, 8 and 9. Here, one can immediately observe that highly expressed isoforms are detectable in all action modes (in particular, for high sequencing depth), the scenario changes dramatically for moderately and lowly expressed ones. The latter have been recognized only in Mode 1 with CA and with sufficient sequencing depth.

The same conclusions apply for **Set-up 2**, comparing Additional file 9: Figure S9, Additional file 10: Figure S10, Additional file 11: Figure S11 and Additional file 12: Figure S12.

It follows that, while it is intriguing to promise to reconstruct the whole transcriptome using only RNA-seq data without any information about annotation, researchers have to be very careful in faithfully believe to the results obtained in Mode 3 (using current experimental protocols and computational methodologies analogues to those considered in the present work). As a consequence, for complex genomes, they should consider to complement the results with other sources of either experimental or computational evidence.

#### Effect of the annotation

In order to evaluate the effect of the annotation, in Figures 2, 3, 4 and 5 precision and recall have to be compared between the two horizontal blocks in each panel A,B,C e D of the same figure (where the upper row refers to CA and the lower row refers to IA) limiting the attention to Mode 1 and Mode 2. While, in Figures 6, 7, 8 and 9 and in Additional file 1: Figure S1, Additional file 2: Figure S2, Additional file 3: Figure S3 and Additional file 4: Figure S4, panels A have to be compared with the corresponding panels B; and panels C with the corresponding panels D.

This effect is very important considering that CA is usually unknown in a real experiment. Therefore, results obtained with CA represent a sort of optimal performance. Results obtained under IA can be seen as a measure of what one can reach with the current knowledge of the Biology.

As expected, moving from CA to IA, regardless the type of alignment, the performances degrade, with a more evident loss in Mode 1 with respect to Mode 2. The observed difference in the two modes of action allows us to illustrate the discovery power of methods in Mode 2. To this purpose, Figures 10 and 11 show the number of transcripts that were not present in IA, but are still recovered by methods in Mode 2 driven by IA. As mentioned before, the “recovering" effect is mainly concentrated on isoforms with high or moderate expression.

In general, the best F-measure observed in Mode 1 with CA is about 0.8 for the higher depth at 100 bp PE and it drops to about 0.6 when IA is used; while in Mode 2 the best F-measure is above 0.6 and drops down to 0.6 when IA is used, compare panel A and B of Additional file 1: Figure S1. Obviously, the present difference depends on the closeness between CA and IA.

Inspecting Figures 2, 3, 4 and 5, it is possible to observe that the loss of performance occurs both in terms of precision and recall. While the loss in detection capability of true isoforms is expected with IA, the loss in precision is more devious. It can be justified by the fact that the methods tend to explain all the reads by assigning them to an isoform. Therefore, other isoforms are used to accommodate the differences in fitting and isoforms with few reads (and low expression) are often formed in absence of a sufficiently strong penalty term or of a post-filtering procedure.

Issues apply for **Set-up 2** comparing Additional file 9: Figure S9, Additional file 10: Figure S10, Additional file 11: Figure S11 and Additional file 12: Figure S12, where IA was randomly generated from CA. Therefore, the main conclusion does not depend strongly on the precise content of IA. As a conclusion, we want to stress that the main limit of methods in Mode 2 is their inadequacy in recovering lowly expressed isoforms, unless already annotated in IA. On the contrary, such methods are able in detecting both moderately and highly expressed isoforms in absence of their annotation as well.

#### Effect of library type

In order to evaluate the effect of the library type, we have to compare PE results with SE results. Such comparison was carried out in **Set-up 1**, for the read length 100 bp and for all depths. Therefore, for precision and recall we have to compare the corresponding panels of Figures 2 and 6 with Figures 5 and 10, respectively; for F-measure we have to compare Additional file 1: Figure S1 with Additional file 4: Figure S4; for number of TP and TN we have to compare Additional file 5: Figure S5 with Additional file 6: Figure S6 for the highest depth (20M) and Additional file 7: Figure S7 with Additional file 8: Figure S8 for the lowest depth (0.25M).

We observe that PE reads show better indexes with respect to SE reads at the same depth (at the price of higher experimental cost, not evaluated here). However, the differences are almost negligible in Mode 1 (in particular, at high coverage), they are relatively small for methods in Mode 2 when CA is provided. The gap increases in Mode 2 when IA is provided. In this case, Figures 10 and 11 show that PE reads allow to correctly detect more isoforms that were not present in IA. The gain is small for high depth, it becomes more evident for low depth. Advantages of using PE is also evident in Mode 3. To better evaluate the differences, we observe that 20M 100 bp-SE allows to map 13918 junctions when CA is provided and 13564 junctions in absence of annotation; 0.25M 100 bp-SE allows to map 8086 junctions when CA is provided and 6851 in absence of annotation.

As a conclusion, we can underline that the main advantage of using PE with respect to SE is in the better capability to recover novel isoforms when not provided in the annotation. On the other hand, we also observe that 100 bp-SE are quite long reads, with short SE reads the advantages of PE are more pronounced.

#### Effect of isoform abundance

In order to evaluate the effect of isoform abundance, we have to inspect Figures 6, 7, 8 and 9 that provide a deeper insight about the recall illustrated in Figures 2, 3, 4 and 5. The index is now expanded into three rows depending on the level of expression of the corresponding true isoforms. From the figures, we can see that the capability in isoform detection strongly depends on their expression levels. In general, highly expressed isoforms are easily detected by methods in Mode 1, while methods in Mode 2 and Mode 3 show a lower (but still acceptable) detection capabilities. On the contrary, moderately and lowly expressed isoforms are detected well, or at least with an acceptable rate, in Mode 1. However, they are not well identified in Mode 2, and often completely lost in Mode 3. Integrating such observation with Figures 10 and 11, we observe that lowly expressed isoforms are mostly detected if they are provided in the annotation.

Additional file 11: Figure S11 and Additional file 12: Figure S12 illustrate the recall with respect to the isoform abundance class in **Set-up 2**, providing analogous conclusions.

#### Effect of sequencing depth

In order to evaluate the effect of sequencing depth, we have to compare different bars of the same colour in each block and methods of panels of Figures 2, 3, 4, 5, 6, 7, 8 and 9, and the behaviour of each coloured line in the F-measure reported in Additional file 1: Figure S1, Additional file 2: Figure S2, Additional file 3: Figure S3 and Additional file 4: Figure S4.

In most cases, the performance gets worse with the decrease in sequencing depth, but the loss is less evident than what one can expect. In particular, it is almost negligible for methods in Mode 1 with CA and it appears more evident for methods in Mode 2 or 3. The gap increases for data driven alignment and in absence of CA. Indeed, when the depth increases we observed less precision and simultaneously a higher recall. The loss in precision can be explained by the large number of FP isoforms, often with low expression values. More in general, we noticed that as far as a minimum level of depth is reached (in the case of **Set-up 1** such level is estimated in about 1M for PE) then further increases of the depth only play a trade-off role between the observed precision and recall without impacting the overall global performance. Conversely, below the saturation level the global performance drops down.

In a similar way, comparing Additional file 5: Figure S5, Additional file 6: Figure S6 and Additional file 7: Figure S7 and Additional file 6: Figure S6, Additional file 7: Figure S7 and Additional file 8: Figure S8, it is possible to see the benefit in the total number of correctly identified isoforms when increasing the depth from the extreme conditions of 0.25M to 20M.

#### Effect of read length

In order to evaluate the effect of read length, we have to compare precision and recall in Figure 2 (100 bp-PE) with Figures 3 and 4 (75 bp-PE and 50 bp-PE, respectively) and Additional file 1: Figure S1 with Additional file 2: Figure S2 and Additional file 3: Figure S3 in terms of F-measure. We found, as expected, that long reads are preferable to short ones. In particular, we observed an overall loss of performance both in terms of recall and precision. We quantified in about 5% the loss in performance in term of F-measure for methods in Mode 1 (the best performance achieved by RSEM with CA is about 0.78 for 100 bp-PE and becomes 0.75 for 75 bp-PE and 0.73 for 50 bp-PE). A more significant loss was observed when executing methods in Modes 2/3, especially at low depth.

We also observe that in our experimental design short reads are obtained by trimming the long ones. Therefore, short reads generated in this way have a slightly better quality with respect to those (of the same length) generated following the error profile. As a consequence, we expect that the real difference between short and long reads could be slightly larger than the one we have reported.

Additional file 9: Figure S9 and Additional file 10: Figure S10 provide analogous insights for **Set-up 2**.

### Isoform estimation

Here, we illustrate the results for estimation error indexes *E*_1_, *E*_2_ and *E*_3_. Additional file 15: Table S1, Additional file 16: Table S2, Additional file 17: Table S3, Additional file 18: Table S4, Additional file 19: Table S5, Additional file 20: Table S6, Additional file 21: Table S7, Additional file 22: Table S8, Additional file 23: Table S9, Additional file 24: Table S10, Additional file 25: Table S11, Additional file 26: Table S12, Additional file 27: Table S13, Additional file 28: Table S14 Additional file 29: Table S15 and Additional file 30: Table S16 show errors statistics (median, 3rd quartile and maximum value) for the indexes in **Set-up 1**. In particular, the group of tables in Additional file 15: Table S1, Additional file 16: Table S2, Additional file 17: Table S3, Additional file 18: Table S4, Additional file 19: Table S5, Additional file 20: Table S6, Additional file 21: Table S7 and Additional file 22: Table S8 illustrate the results for PE, and those in Additional file 23: Table S9, Additional file 24: Table S10, Additional file 25: Table S11, Additional file 26: Table S12, Additional file 27: Table S13, Additional file 28: Table S14 Additional file 29: Table S15 and Additional file 30: Table S16 for SE. Within each group, the two most extreme depths are shown (i.e., 20M and 0.25M). Furthermore, the tables provide results either for annotation driven alignment (CA and IA) and for data driven alignment. Additional file 31: Table S17, Additional file 32: Table S18, Additional file 33: Table S19 and Additional file 34: Table S20 refer to **Set-up 2**. In particular, Additional file 31: Table S17 and Additional file 32: Table S18 describe the results for 60M 75 bp-PE (annotation driven alignment). The analogous cases for data driven alignment are shown in Additional file 33: Table S19 and Additional file 34: Table S20. In order to investigate the performance of the methods in correctly estimating the isoform abundances, the attention have to be mainly focused on the qualitative aspects related to error distributions rather that on the actual values shown in the tables.

Overall, both set-ups show a similar behaviour for all error types. In fact, all methods produce errors that have a strongly asymmetric skewed distribution with a long right tail. This means that they fail to estimate a (significant) fraction of isoforms (see results for the 3rd quartile and the maximum observed value). Moreover, within the same table, the same index is also different (sometimes of order of magnitude) with respect to methods and mode of actions. Differences are observed, not only with respect to the most extreme value, but also with respect to the median and the 3rd quartile. This means that the methods may provide very different results on a large fraction of isoforms. In brief, all tables indicate that the problem of obtaining reliable estimates is still open. The loss of performance is not completely surprising, since estimation is carried out after isoform detection, without explicitly consider the uncertainty due to the identification step. The latter statement is clear when we compare the same method under different modes of action. In such cases, each method uses an analogous statistical procedure for estimating the abundances. However, the inference is carried out on different sets of isoforms and can produce different estimates. To mitigate such drawbacks confidence intervals for the estimates could be more reliable than point estimates.

More in detail, looking at the 3rd quartile in Additional file 15: Table S1 (i.e., 20M 100 bp-PE CA driven alignment), the best result for *E*_1_ oscillates between 0.27–0.29 (CEM and Cufflinks Mode 3, RSEM), for *E*_2_ is about 0.005 (Cufflinks, Mode 1), and for *E*_3_ is between 0.22–0.25 (RSEM, CEM Mode 1).

The low 3rd quartiles observed for *E*_3_ and *E*_2_ confirm that problems arise mainly with lowly expressed isoforms, that are either not detected or not set to zero by filtering or penalization procedures. On the contrary, the large extreme values observed for *E*_2_ and *E*_3_ indicate that the corresponding methods can completely fail (of several order of magnitude) some estimates. However, such failures are limited to few units when the corresponding 3rd quartile is low, being larger when the right tail of the distribution becomes heavier.

More attention requires the (relative) error *E*_1_, whose 3rd quartile is at least about 0.27–0.29 in Additional file 15: Table S1, (with the median value of about 0.07–0.15). This means that, even when we are able to correctly detect the presence of the isoforms, in more than 50% of the cases the estimation error is about 10% of the true value. Larger values for the median or the 3rd quartile indicate a worse performance.

We stress that Additional file 15: Table S1 illustrates (in principle) the most favourable condition. Comparing it with Additional file 17: Table S3 (i.e., 0.25M 100 bp-PE CA driven alignment), it is possible to evaluate the effect of the depth when the annotation is CA. Whereas precision and recall for methods in Mode 1 were not significantly influenced, the error indexes did. In particular, the largest differences are observed for *E*_2_ and *E*_3_. In general, at low depth the quality of the estimates is quite poor. Analogous considerations hold considering SE instead of PE, see Additional file 23: Table S9 and Additional file 15: Table S11, for the cases 20M SE and 0.25M SE, respectively.

The influence of annotation can be deduced comparing Additional file 15: Table S1 and Additional file 17: Table S2 (obtained using IA). Analogous comparison can be carried out for all pairs of corresponding tables (CA vs IA). In such cases the differences are larger, due to the fact that methods are not able to identify all isoforms (in particular those not provided in IA).

Additional file 31: Table S17, Additional file 32: Table S18, Additional file 33: Table S19 and Additional file 34: Table S20 drive to similar considerations for **Set-up 2** with more skewed error distributions.

### Considerations about the computational cost

Since the methods were implemented in different languages and ran in different environments, we found that a technical comparison of execution time was not fair. Therefore, we briefly reports only qualitative considerations. RSEM and Cufflinks (in all modes of action) are sufficiently fast, CEM (in all modes of action) is the fastest. On the contrary, iReckon is quite slow with respect to the others and also produces very large temporary files. Current implementation of SLIDE is too slow to be completed in **Set-up 2** in a reasonable amount of time, and therefore was considered only in **Set-up 1**.

## Conclusions

Our results show that algorithms have good or acceptable performance in detecting the presence of the isoforms (recall) when the annotation is provided, even if incomplete (Mode 1 and Mode 2, with both CA and IA). On the contrary, the data driven methods (Mode 3) are still not satisfactory, also when the reads are carefully aligned using the annotation during the mapping phase. Results obtained in Mode 3 are in agreement with what observed in [24].

The performance of all methods is strongly influenced by the strength of the isoforms. Highly and moderately expressed isoforms are identified with good accuracy. On the contrary, lowly expressed isoforms are still problematic, also when the exact annotation is provided. In particular, we observed that lowly expressed isoforms that are not present in the annotation, are not recovered using methods in Mode 2. Conversely, Mode 2 approaches are able to recover part of moderately and most of highly expressed transcripts also when not provided in the annotation.

Results improve by increasing the sequencing depth and the read-length. However, for the depth there is a saturation limit -in current computational methodologies- that does not allow to achieve optimal reconstruction even at high coverage. In fact, when the coverage increases, the number of TP increase as expected, but also the number of FP increases. In particular, we noticed that some methods tend to identify several (novel and annotated) isoforms as present at very low expression levels. In most cases such isoforms are FP, as a consequence precision is often not satisfactory.

Realistic estimation of isoforms abundance is also very problematic. Similar to [24] our results show that the estimation error is very skewed. The error distribution up to the third quartile is acceptable at least for E2 (connected to FP) and E3 (connected to FN), with a very long right tail. On the contrary, the distribution of the (relative) error E1 shows that more than 50% of isoforms are estimated with more than 10% of error. To this purpose, we should consider that estimation of isoform abundances is not easier than isoform identification. In fact, the estimates are often obtained for the set of isoforms that have been previously identified. However, methods usually do not take into account such uncertainty. As a consequence, the performance can be much worse. Therefore, we conclude that the estimation of the correctly identified isoforms is still challenging.

As observed by [24], the complexity of higher eukaryotic genomes, such as the human one, imposes severe limitations to the performance of all quantification and estimation methods, that are likely to remain limiting factors for the analysis of current-generation RNA-seq experiments. Such limitations can be partially solved providing existing annotations, but more in general require the development of further research and techniques from both methodological and experimental point of views.

Finally, it should be noted that all methods considered here and in [24] work with a single RNA-seq sample. Recent works [49,50] propose to use a multiple-sample approach to achieve a more precise identification and estimation of isoform expression. The availability of such type of approaches, whose performances have to be further validated, seems to indicate that future studies have to investigate a larger variety of (homogeneous) samples at a lower depth per sample to obtain more confident transcript predictions, see [50].

## Competing interests

The authors declare that they have no competing interests.

## Authors’ contributions

All authors participated in conceiving the analysis and setting-up the code for the comparisons and writing the manuscript. All authors also read and approved the submitted manuscript.

## Supplementary Material

Additional file 1 **Figure S1.** F-measure in Set-up 1 for 100 bp-PE. Panels **A** (upper left) and **B** (upper right) depict F-measure versus the sequencing depth for each compared method when the alignment is annotation driven using CA and IA, respectively. Panels **C** (bottom left) and **D** (bottom right) are analogous to Panels **A** and **B**, when the alignment is data driven. The figure refers to Set-up 1 and 100 bp-PE. Within each panel, methods in Mode 1 are depicted with continuous line, methods in Mode 2 with dashed line, methods in Mode 3 with dotted line. When the alignment is annotation driven, the same annotation provided during the alignment was used for Mode 1 and 2. Click here for file

Additional file 2 **Figure S2.** F-measure in Set-up 1 for 75 bp-PE. Analogous to Additional file 1: Figure S1, but for Set-up 1 and 75 bp-PE. Click here for file

Additional file 3 **Figure S3.** F-measure in Set-up 1 for 50 bp-PE. Analogous to Additional file 1: Figure S1, but for Set-up 1 and 50 bp-PE. Click here for file

Additional file 4 **Figure S4.** F-measure in Set-up 1 for 100 bp-SE. Analogous to Additional file 1: Figure S1, but for Set-up 1 and 100 bp-SE. Click here for file

Additional file 5 **Figure S5.** True Positives and False Positives in Set-up 1 for 20M 100 bp-PE. Panels **A** (upper left) and **B** (upper right) depict TP (coral) and FP (aquamarine) bars for the compared methods when the alignment is annotation driven (CA and IA, respectively). Panels **C** (bottom left) and **D** (bottom right) are analogous to Panels **A** and **B**, when the alignment is data driven. The figure refers to Set-up 1 and 20M 100 bp-PE. The true number of expressed transcripts (i.e., 3726) is added as dashed horizontal line to each panel. The difference between the TP and the horizontal line represents the FN. Click here for file

Additional file 6 **Figure S6.** True Positives and False Positives in Set-up 1 for 20M 100 bp-SE. Analogous to Additional file 5: Figure S5, but for Set-up 1 and 20M 100 bp-SE. Click here for file

Additional file 7 **Figure S7.** True Positives and False Positives in Set-up 1 for 0.25M 100 bp-PE. Analogous to Additional file 5: Figure S5, but for Set-up 1 and 0.25M 100 bp-PE. Click here for file

Additional file 8 **Figure S8.** True Positives and False Positives in Set-up 1 for 0.25M 100 bp-SE. Analogous to Additional file 5: Figure S5, but for Set-up 1 and 0.25M 100 bp-PE. Click here for file

Additional file 9 **Figure S9.** Precision and Recall bar-plot in Set-up 2 for 75 bp-PE. Analogous to Figure 2, but for Set-up 2 for 60M 75 bp-PE. Click here for file

Additional file 10 **Figure 10.** Precision and Recall bar-plot in Set-up 2 for 50 bp-PE. Analogous to Figure 2, but for Set-up 2 for 60M 50 bp-PE. Click here for file

Additional file 11 **Figure S11.** Recall bar-plot versus isoform abundance in Set-up 2 for 60M 75 bp-PE. Analogous to Figure 5, but for Set-up 2 for 60M 75 bp-PE. Click here for file

Additional file 12 **Figure S12.** Recall bar-plot versus isoform abundance in Set-up 2 for 60M 50 bp-PE. Analogous to Figure 5, but for Set-up 2 for 60M 50 bp-PE. Click here for file

Additional file 13 **Figure S13.** Precision, Recall and F-measure when introducing thresholds (Set-up 2). Analogous to Figure 12, but for Set-up 2, 60M 75 bp-PE and the alignment with CA. Click here for file

Additional file 14 **Figure S14.** True Positives and False Positives in Set-up 2 for 60M 75 bp-PE. Panels **A** (upper left) and **B** (upper right) depict TP (coral) and FP (aquamarine) bars for the compared methods when the alignment is annotation driven (CA and IA, respectively). Panels **C** (bottom left) and **D** (bottom right) are analogous to Panels **A** and **B**, when the alignment is data driven. The figure refers to Set-up 2 and 60M 75 bp-PE. The true number of expressed transcripts (i.e., 17032) is added as dashed horizontal line to each panel. The difference between the TP and the horizontal line represents the FN. Click here for file

Additional file 15 **Table S1.** E1, E2 and E3 in Set-up 1 with annotation driven alignment (CA) for 20M 100 bp-PE. Median, 3rd Quartile and maximum value observed for error indexes E1, E2 and E3 in Set-up 1, 20M PE reads of 100 bp. Tables are divided in blocks, where the left block is for methods used in Mode 1, middle block is for methods used in Mode 2, right block is for methods used in Mode 3. Upper rows refer to E1, middle ones refer to E2, bottom ones refer to E3. CA was provided during the alignment. Click here for file

Additional file 16 **Table S2.** E1, E2 and E3 in Set-up 1 with annotation driven alignment (IA) for 20M 100 bp-PE. Analogous to Additional file 15: Table S1, but with IA provided during the alignment. Click here for file

Additional file 17 **Table S3.** E1, E2 and E3 in Set-up 1 with annotation driven alignment (CA) for 0.25M 100 bp-PE. Analogous to Additional file 15: Table S1, but for 0.25M PE reads. Click here for file

Additional file 18 **Table S4.** E1, E2 and E3 in Set-up 1 with annotation driven alignment (IA) for 0.25M 100 bp-PE. Analogous to Additional file 15: Table S1, but for 0.25M PE reads and with IA provided during the alignment. Click here for file

Additional file 19 **Table S5.** E1, E2 and E3 in Set-up 1, with data driven alignment and CA for 20M 100 bp-PE. Analogous to Additional file 15: Table S1, but with data driven alignment. CA was provided in Mode 1 and Mode 2. Click here for file

Additional file 20 **Table S6.** E1, E2 and E3 in Set-up 1, with data driven alignment and IA for 20M 100 bp-PE. Analogous to Additional file 15: Table S1, but with data driven alignment. IA was provided in Mode 1 and Mode 2. Click here for file

Additional file 21 **Table S7.** E1, E2 and E3 in Set-up 1, with data driven alignment and CA for 0.25M 100 bp-PE. Analogous to Additional file 19: Table S5, but for 0.25M PE reads. Click here for file

Additional file 22 **Table S8.** E1, E2 and E3 in Set-up 1, with data driven alignment and IA for 0.25M 100 bp-PE. Analogous to Additional file 20: Table S6, but for 0.25M PE reads. Click here for file

Additional file 23 **Table S9.** E1, E2 and E3 in Set-up 1 with annotation driven alignment (CA) for 20M 100 bp-SE. Analogous to Additional file 15: Table S1, but for 20M SE reads. Click here for file

Additional file 24 **Table S10.** E1, E2 and E3 in Set-up 1 with annotation driven alignment (IA) for 20M 100 bp-SE. Analogous to Additional file 17: Table S2, but for 20M SE reads. Click here for file

Additional file 25 **Table S11.** E1, E2 and E3 in Set-up 1 with annotation driven alignment (CA) for 0.25M 100 bp-SE. Analogous to Additional file 17: Table S3, but for 0.25M SE reads. Click here for file

Additional file 26 **Table S12.** E1, E2 and E3 in Set-up 1 with annotation driven alignment (IA) for 0.25M 100 bp-SE. Analogous to Additional file 18: Table S4, but for 0.25M SE reads. Click here for file

Additional file 27 **Table S13.** E1, E2 and E3 in Set-up 1, with data driven alignment and CA for 20M 100 bp-SE. Analogous to Additional file 19: Table S5, but for 20M SE reads. Click here for file

Additional file 28 **Table S14.** E1, E2 and E3 in Set-up 1, with data driven alignment and IA for 20M 100 bp-SE. Analogous to Additional file 20: Table S6, but for 20M SE reads. Click here for file

Additional file 29 **Table S15.** E1, E2 and E3 in Set-up 1, with data driven alignment and CA for 0.25M 100 bp-SE. Analogous to Additional file 21: Table S7, but for 025M SE reads. Click here for file

Additional file 30 **Table S16.** E1, E2 and E3 in Set-up 1, with data driven alignment and IA for 0.25M 100 bp-SE. Analogous to Additional file 22: Table S8, but for 025M SE reads. Click here for file

Additional file 31 **Table S17.** Statistics for E1, E2 and E3 in Set-up 2 with annotation driven alignment (CA) for 60M 75 bp-PE. Analogous to Additional file 15: Table S1, but for Set-up 2, 60M 75 bp-PE. Click here for file

Additional file 32 **Table S18.** Statistics for E1, E2 and E3 in Set-up 2 with annotation driven alignment (IA) for 60M 75 bp-PE. Analogous to Additional file 17: Table S2, but for Set-up 2, 60M 75 bp-PE. Click here for file

Additional file 33 **Table S19.** Statistics for E1, E2 and E3 in Set-up 2 with data driven alignment and CA for 60M 75 bp-PE. Analogous to Additional file 19: Table S5, but for Set-up 2, 60M 75 bp-PE. Click here for file

Additional file 34 **Table S20.** Statistics for E1, E2 and E3 in Set-up 2 with data driven alignment and IA for 60M 75 bp-PE. Analogous to Additional file 20: Table S6, but for Set-up 2, 60M 75 bp-PE. Click here for file
