# Supplementary material for: Computational approaches for isoform detection and estimation: good and bad news
Source: BMC Bioinformatics. 2014 May 9;15:135. doi: 10.1186/1471-2105-15-135 (PMC4098781; doi:10.1186/1471-2105-15-135)

PE 100 bp (Set-up 1)

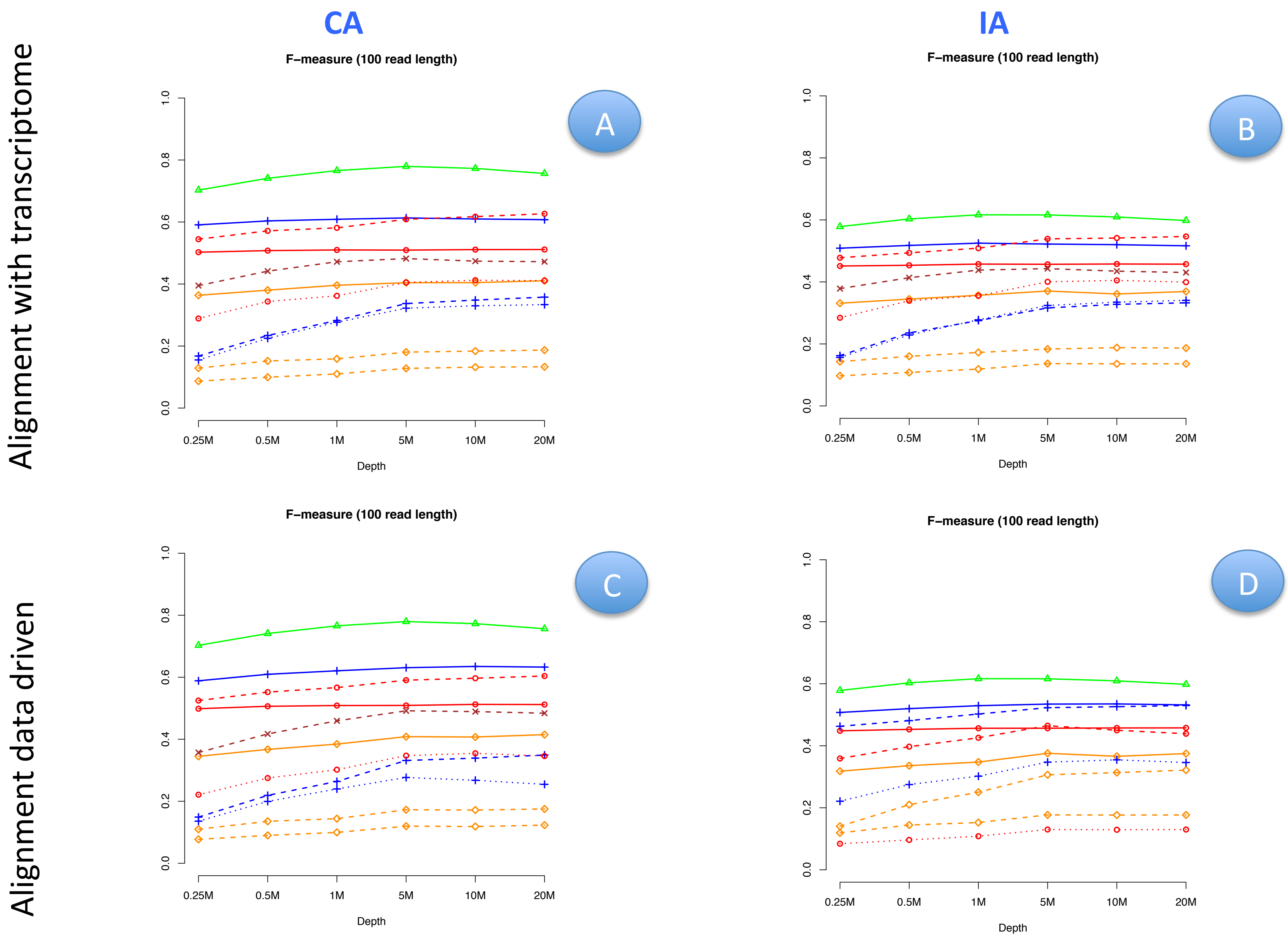

Supplement: Additional file 1 — Figure S1. F-measure in Set-up 1 for 100 bp-PE. Panels A (upper left) and B (upper right) depict F-measure versus the sequencing depth for each compared method when the alignment is annotation driven using CA and IA, respectively. Panels C (bottom left) and D (bottom right) are analogous to Panels A and B, when the alignment is data driven. The figure refers to Set-up 1 and 100 bp-PE. Within each panel, methods in Mode 1 are depicted with continuous line, methods in Mode 2 with dashed line, methods in Mode 3 with dotted line. When the alignment is annotation driven, the same annotation provided during the alignment was used for Mode 1 and 2. [file 1471-2105-15-135-S1.pdf]
