# Supplementary figures and images for: Computational approaches for isoform detection and estimation: good and bad news
Source: BMC Bioinformatics. 2014 May 9;15:135. doi: 10.1186/1471-2105-15-135 (PMC4098781; doi:10.1186/1471-2105-15-135)

PE 75 bp (Set-up 1)

Alignment with transcriptome

Alignment data driven

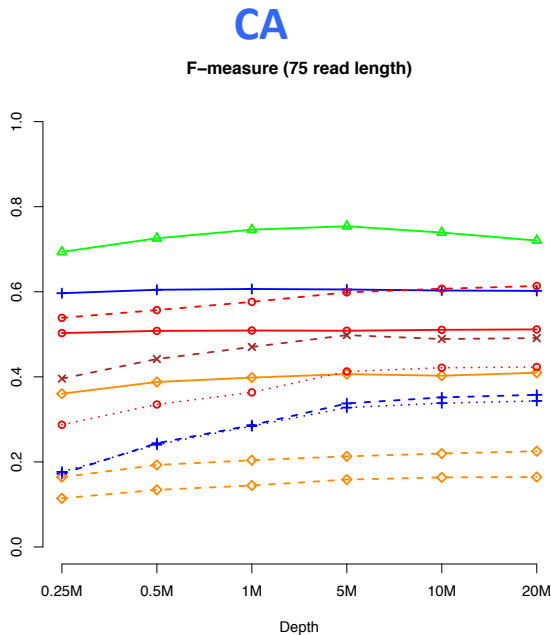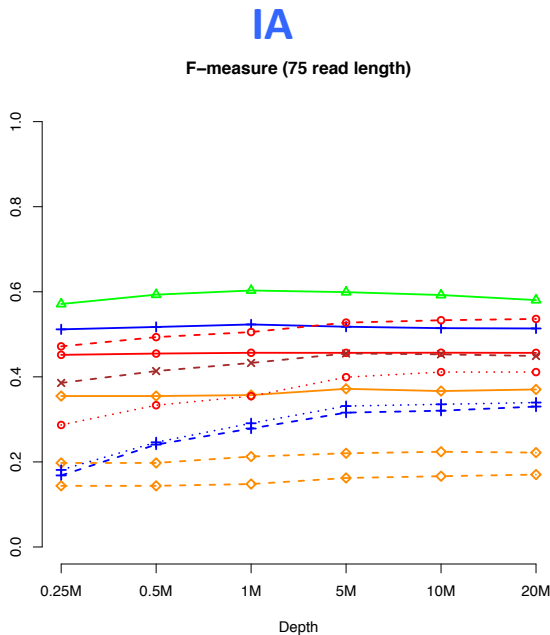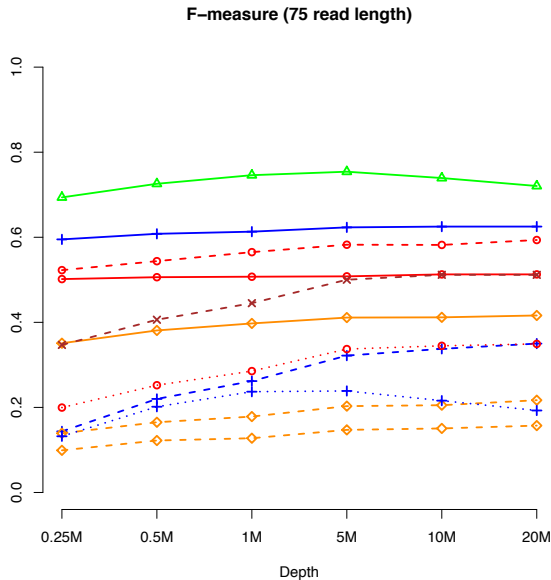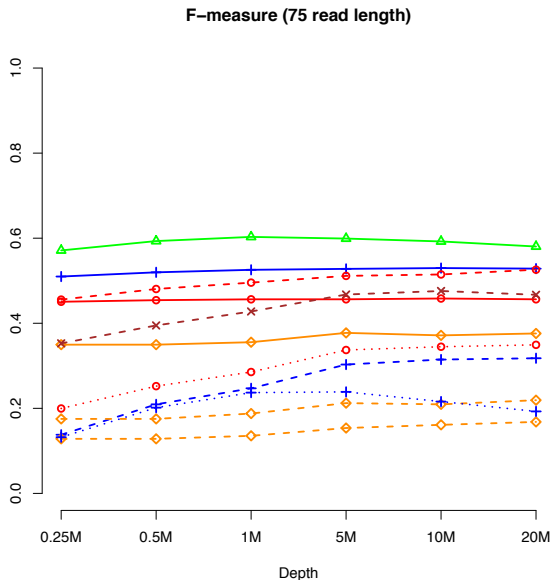

Supplement: Additional file 2 — Figure S2. F-measure in Set-up 1 for 75 bp-PE. Analogous to Additional file 1: Figure S1, but for Set-up 1 and 75 bp-PE. [file 1471-2105-15-135-S2.pdf]

PE 100 bp – 20 M (Set-up 1)

Alignment with transcriptome

CA

IA

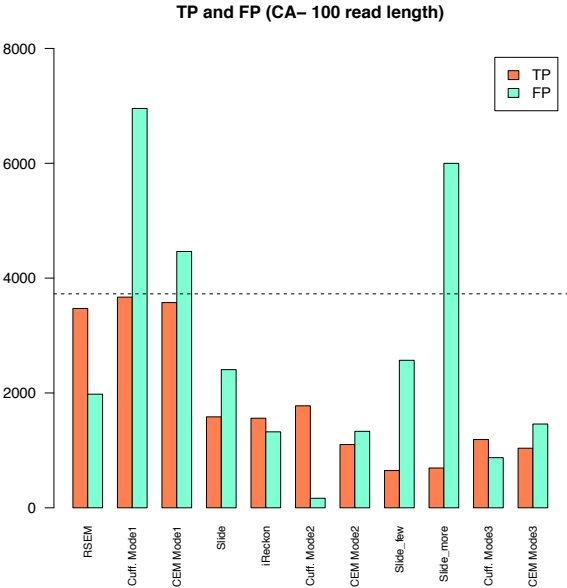

A

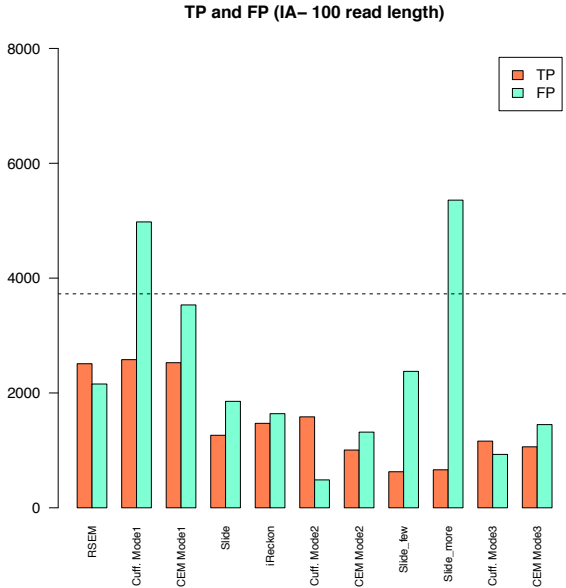

B

Alignment data driven

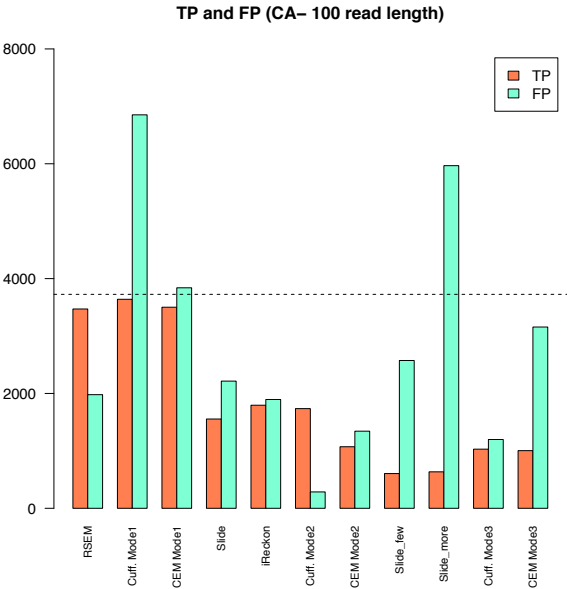

C

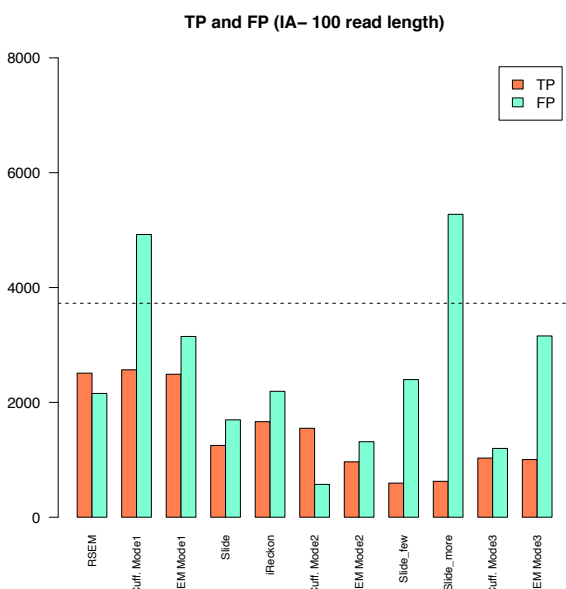

D

Supplement: Additional file 5 — Figure S5. True Positives and False Positives in Set-up 1 for 20M 100 bp-PE. Panels A (upper left) and B (upper right) depict TP (coral) and FP (aquamarine) bars for the compared methods when the alignment is annotation driven (CA and IA, respectively). Panels C (bottom left) and D (bottom right) are analogous to Panels A and B, when the alignment is data driven. The figure refers to Set-up 1 and 20M 100 bp-PE. The true number of expressed transcripts (i.e., 3726) is added as dashed horizontal line to each panel. The difference between the TP and the horizontal line represents the FN. [file 1471-2105-15-135-S5.pdf]

# PE 75 bp (Set-up 2)

Alignment with transcriptome

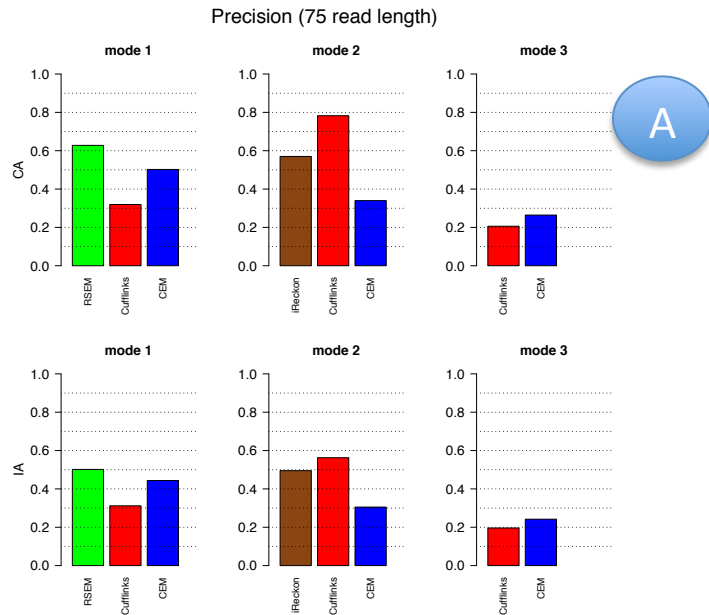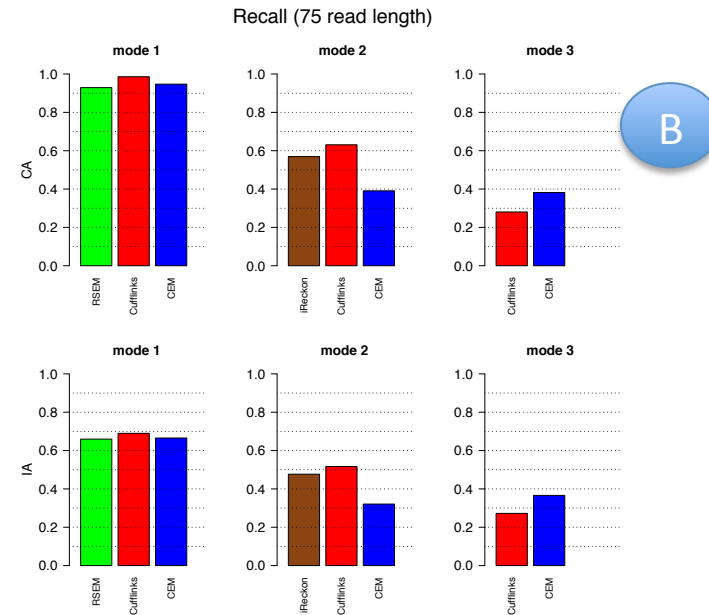

Alignment data driven

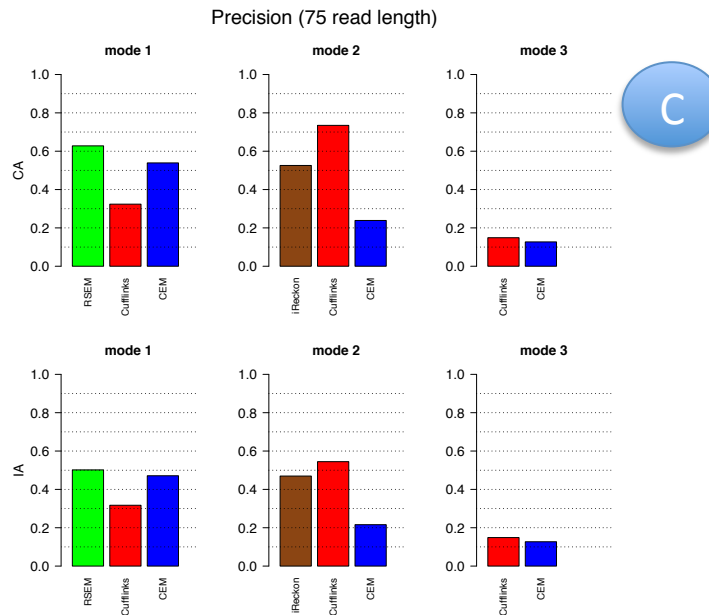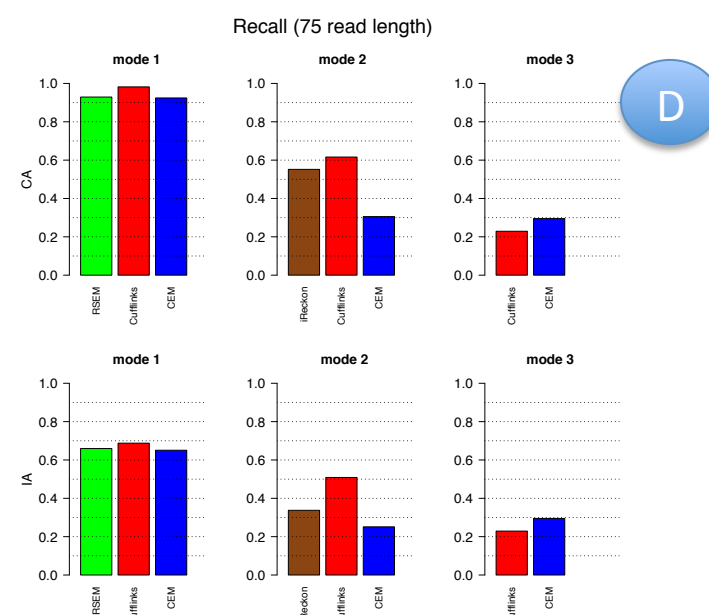

Supplement: Additional file 9 — Figure S9. Precision and Recall bar-plot in Set-up 2 for 75 bp-PE. Analogous to Figure 2, but for Set-up 2 for 60M 75 bp-PE. [file 1471-2105-15-135-S9.pdf]

# Set-up 2, CA, alignment with transcriptome

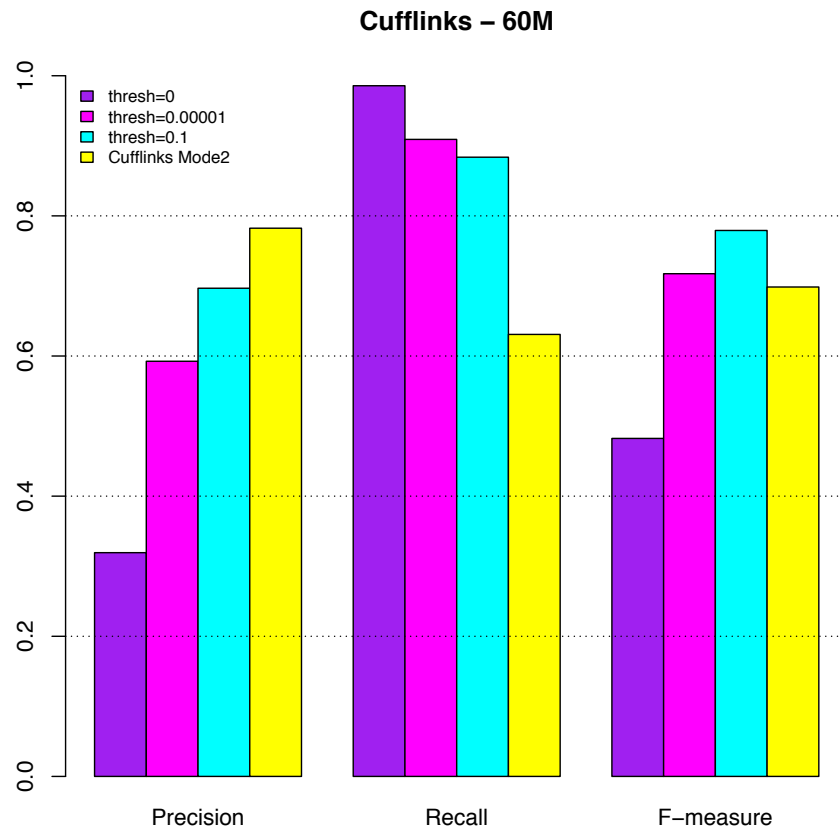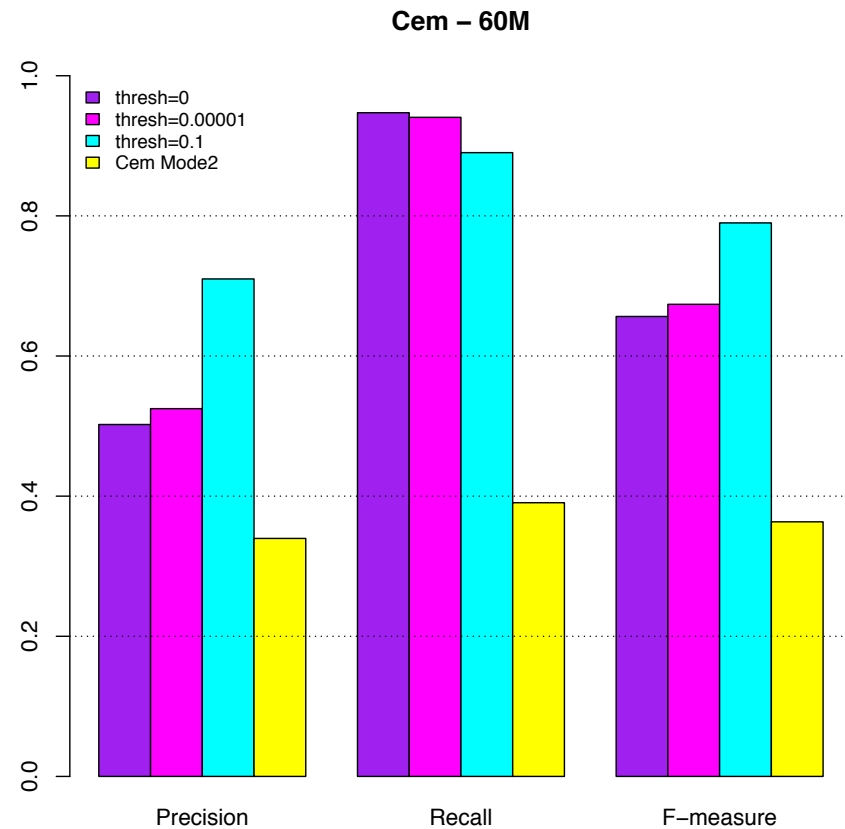

Supplement: Additional file 13 — Figure S13. Precision, Recall and F-measure when introducing thresholds (Set-up 2). Analogous to Figure 12, but for Set-up 2, 60M 75 bp-PE and the alignment with CA. [file 1471-2105-15-135-S13.pdf]
