# Supplementary material for: Computational approaches for isoform detection and estimation: good and bad news
Source: BMC Bioinformatics. 2014 May 9;15:135. doi: 10.1186/1471-2105-15-135 (PMC4098781; doi:10.1186/1471-2105-15-135)

SE 100 bp – 20 M (Set-up 1)

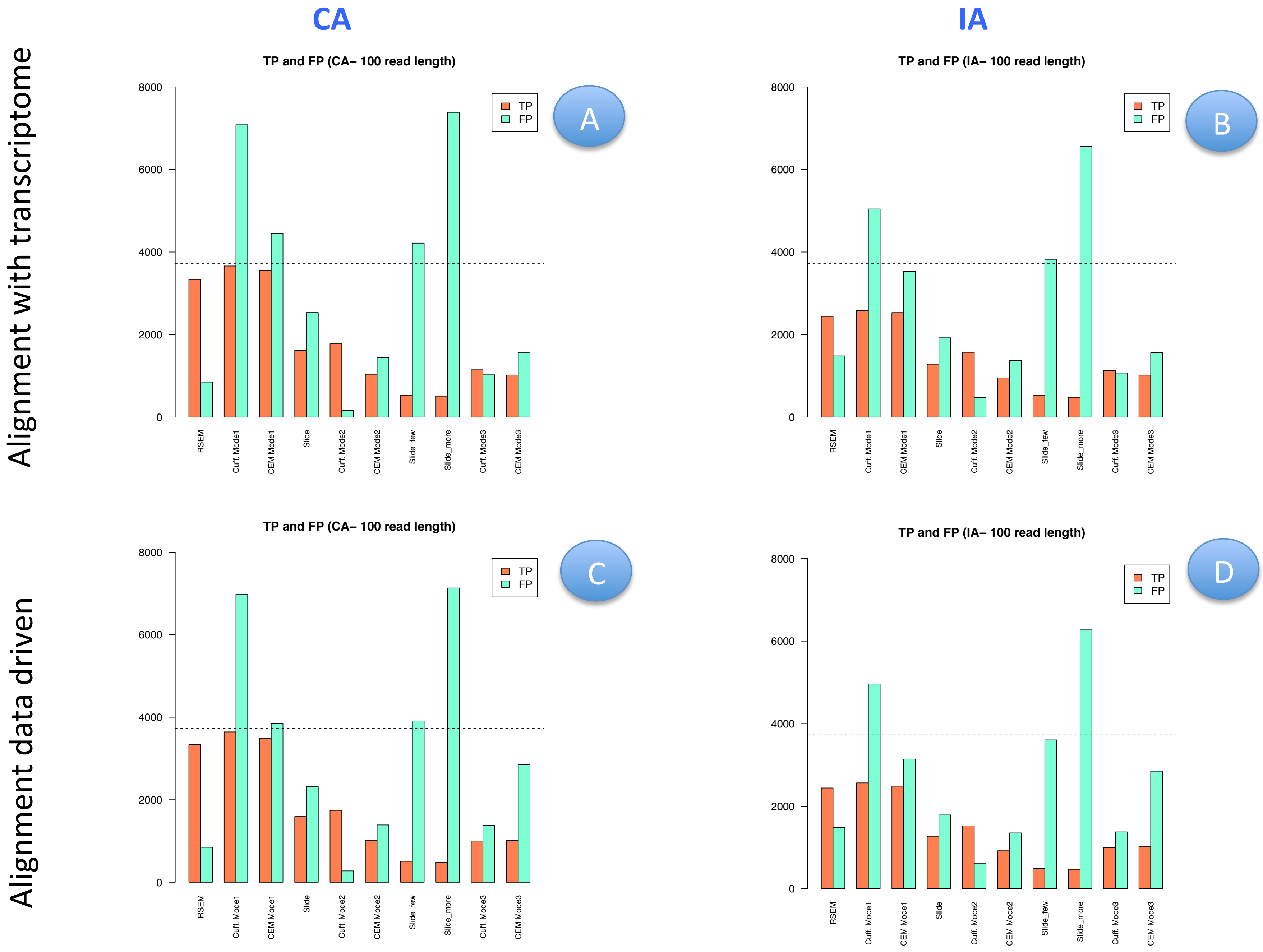

Supplement: Additional file 6 — Figure S6. True Positives and False Positives in Set-up 1 for 20M 100 bp-SE. Analogous to Additional file 5: Figure S5, but for Set-up 1 and 20M 100 bp-SE. [file 1471-2105-15-135-S6.pdf]
