# Supplementary material for: Computational approaches for isoform detection and estimation: good and bad news
Source: BMC Bioinformatics. 2014 May 9;15:135. doi: 10.1186/1471-2105-15-135 (PMC4098781; doi:10.1186/1471-2105-15-135)

PE 50 bp (Set-up 2)

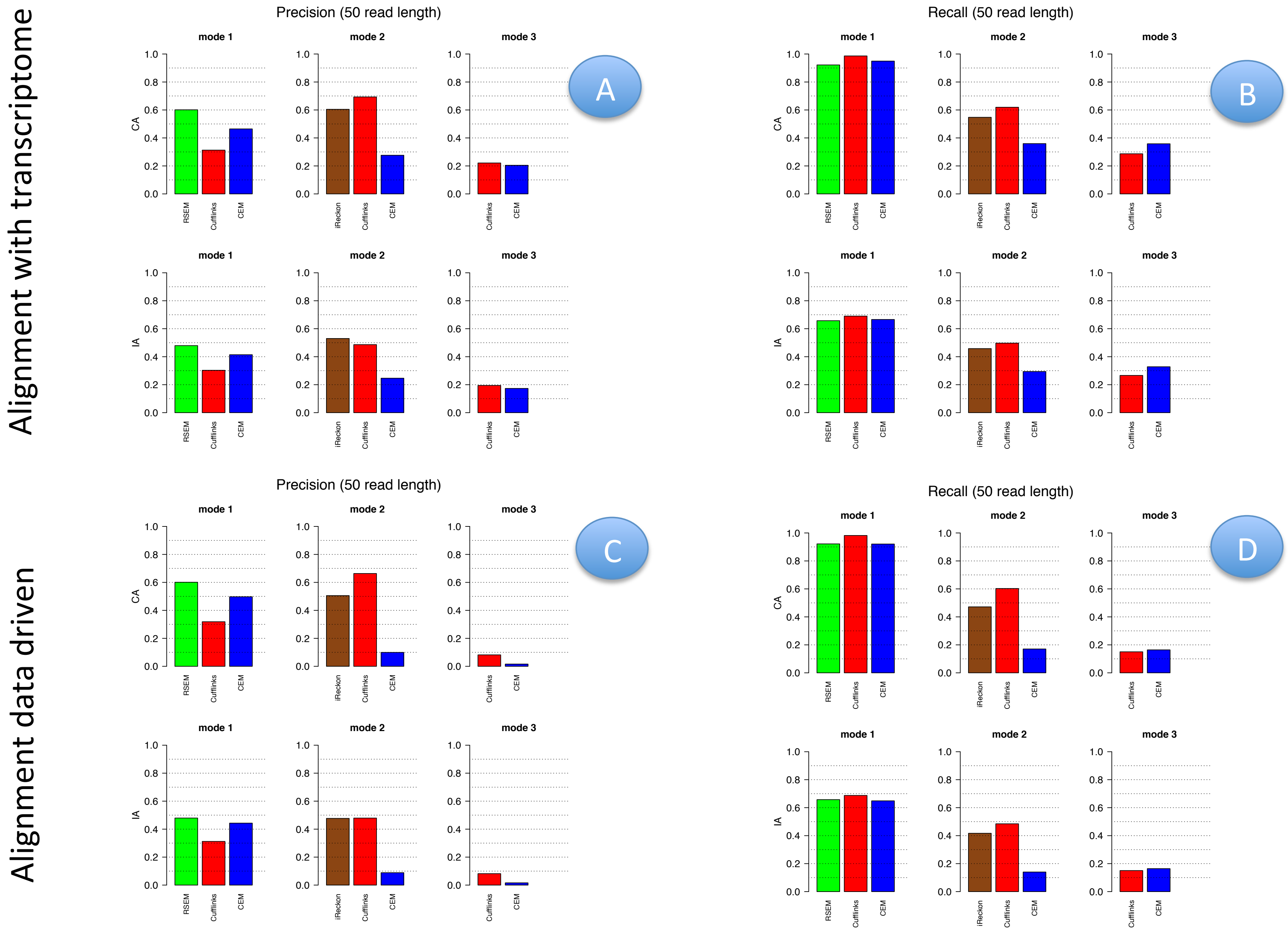

Supplement: Additional file 10 — Figure 10. Precision and Recall bar-plot in Set-up 2 for 50 bp-PE. Analogous to Figure 2, but for Set-up 2 for 60M 50 bp-PE. [file 1471-2105-15-135-S10.pdf]
