# Supplementary material for: Computational approaches for isoform detection and estimation: good and bad news
Source: BMC Bioinformatics. 2014 May 9;15:135. doi: 10.1186/1471-2105-15-135 (PMC4098781; doi:10.1186/1471-2105-15-135)

PE 75 bp – 60 M (Set-up 2)

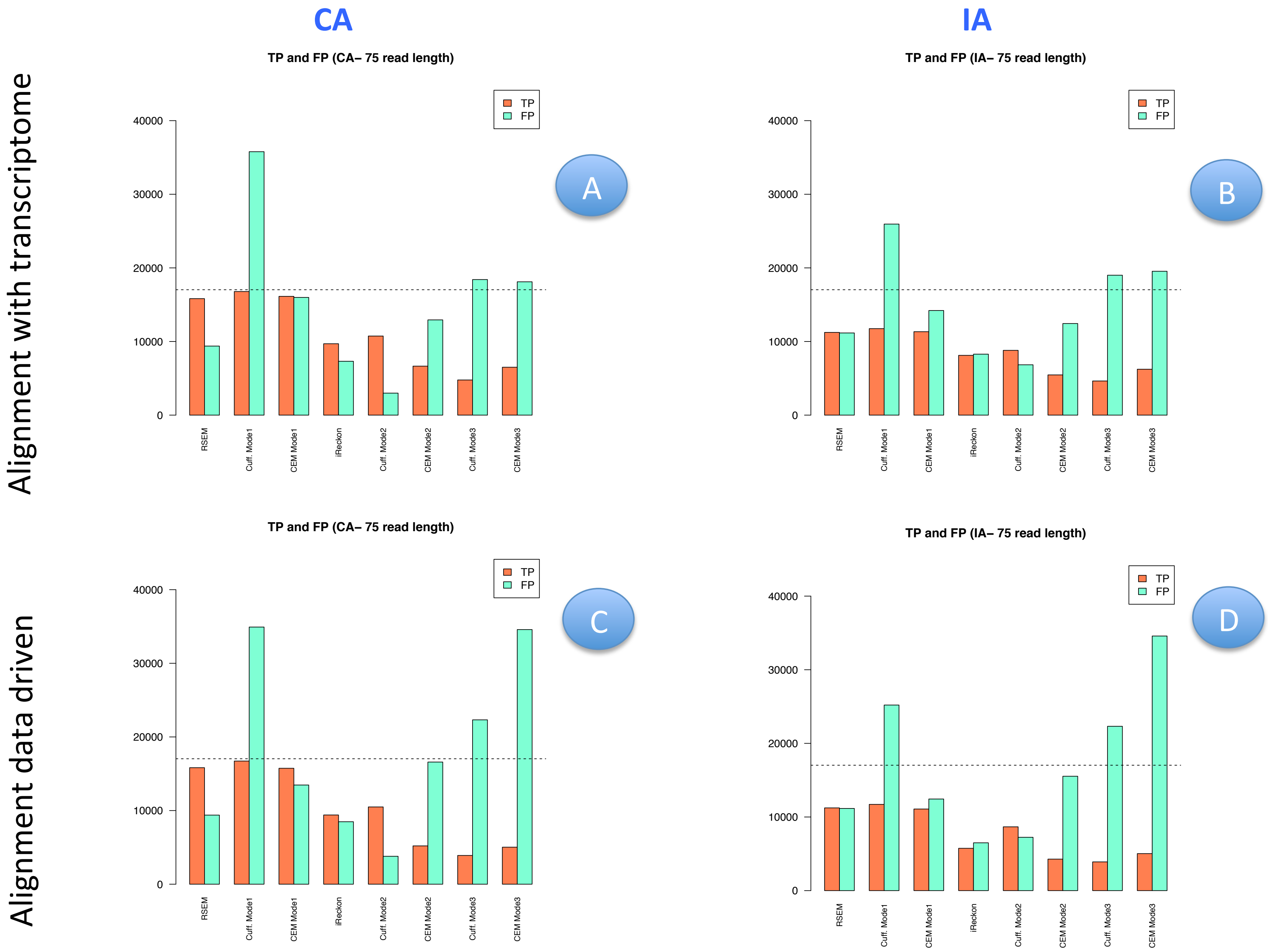

Supplement: Additional file 14 — Figure S14. True Positives and False Positives in Set-up 2 for 60M 75 bp-PE. Panels A (upper left) and B (upper right) depict TP (coral) and FP (aquamarine) bars for the compared methods when the alignment is annotation driven (CA and IA, respectively). Panels C (bottom left) and D (bottom right) are analogous to Panels A and B, when the alignment is data driven. The figure refers to Set-up 2 and 60M 75 bp-PE. The true number of expressed transcripts (i.e., 17032) is added as dashed horizontal line to each panel. The difference between the TP and the horizontal line represents the FN. [file 1471-2105-15-135-S14.pdf]
